# MVP-mediated exosomal sorting of miR-193a promotes colon cancer progression

**DOI:** 10.1038/ncomms14448

**Published:** 2017-02-17

**Authors:** Yun Teng, Yi Ren, Xin Hu, Jingyao Mu, Abhilash Samykutty, Xiaoying Zhuang, Zhongbin Deng, Anil Kumar, Lifeng Zhang, Michael L. Merchant, Jun Yan, Donald M. Miller, Huang-Ge Zhang

**Affiliations:** 1 James Graham Brown Cancer Center, University of Louisville, Louisville, Kentucky 40202, USA; 2 Department of Breast and Thyroid Surgery, Huai'an First People's Hospital, Huai'an, Jiangsu 223001, China; 3 Program in Biostatistics, Bioinformatics and Systems Biology, The University of Texas Graduate School of Biomedical Sciences at Houston, Houston, Texas 77030, USA; 4 Department of Genomic Medicine, The University of Texas MD Anderson Cancer Center, Houston, Texas 77030, USA; 5 Kidney Disease Program and Clinical Proteomics Center, University of Louisville, Louisville, Kentucky, USA; 6 Robley Rex VA Medical Center, Louisville, Kentucky 40206, USA

## Abstract

Exosomes are emerging mediators of intercellular communication; whether the release of exosomes has an effect on the exosome donor cells in addition to the recipient cells has not been investigated to any extent. Here, we examine different exosomal miRNA expression profiles in primary mouse colon tumour, liver metastasis of colon cancer and naive colon tissues. In more advanced disease, higher levels of tumour suppressor miRNAs are encapsulated in the exosomes. miR-193a interacts with major vault protein (MVP). Knockout of MVP leads to miR-193a accumulation in the exosomal donor cells instead of exosomes, inhibiting tumour progression. Furthermore, miR-193a causes cell cycle G1 arrest and cell proliferation repression through targeting of Caprin1, which upregulates Ccnd2 and c-Myc. Human colon cancer patients with more advanced disease show higher levels of circulating exosomal miR-193a. In summary, our data demonstrate that MVP-mediated selective sorting of tumour suppressor miRNA into exosomes promotes tumour progression.

## Abstract (web summary)

Exosomes are involved in the development of metastasis but how their composition is regulated is not well known. Here the authors propose that major vault protein-dependent loading of miR-193a into exosomes could be a general mechanism by which cancer cells get rid of oncosuppressor miRNAs.

Cancer cells secrete extracellular vesicles (EVs) that promote cancer progression^1^,^2^,^3^,^4^, and these EVs contain markers for potential cancer diagnosis and prognosis^5^,^6^. A complete working model of EV-mediated biological effects has not been demonstrated in a fully physiological *in vivo* context and is urgently needed for understanding the *in vivo* fate of cancer EVs. Most studies published thus far analyse the function of EV populations isolated from the supernatants of cultured cells^7^,^8^,^9^. One of the challenges of this experimental methodology is whether EV secretion *in vitro* by tumour cells is capable of achieving the necessary result *in vivo*. A number of approaches have been undertaken to address this challenge by either interfering with *in vivo* EV biogenesis in cancer cells with siRNA knockdown^10^,^11^ or with chemical inhibitors^12^ to inhibit EV release. However, EVs, including exosomes, are released from many different types of cells, including cancer cells as well as non-cancer cells. Without a known cancer exosome-specific marker, we cannot define the biological effect of cancer cell-derived exosomes from non-cancer cell-derived exosomes in an *in vivo* model.

Recently, modifications of EVs that allow tracking of their target cells *in vivo* have been reported^13^,^14^,^15^. In the current study, we generated a stable colon cancer cell line utilizing a vector^16^ expressing a luciferase protein fused to a biotin acceptor peptide with a transmembrane (TM) domain of platelet-derived growth factor. The fused proteins with a TM domain localize in the plasma membrane as well as in secreted EVs (ref. 16). Therefore, exosomes released from this stable colon cancer cell line are biotinylated and can be isolated from mixed EVs with streptavidin-coated beads, allowing for their miRNA profile to be further analysed. The tumour tissue-derived exosome miRNA profile is detected in the exosomes from peripheral blood of tumour-bearing mice but not naive mice. In this study, as a proof of concept, we hypothesize that tumour exosomes selectively sort tumour suppressor miRNA into exosomes, whereas oncogenic miRNA is kept in the tumour cell regardless of the level of miRNA expressed in the cell.

## Results

### Isolation and characterization of tumour-specific exosomes

**Figure 1:**
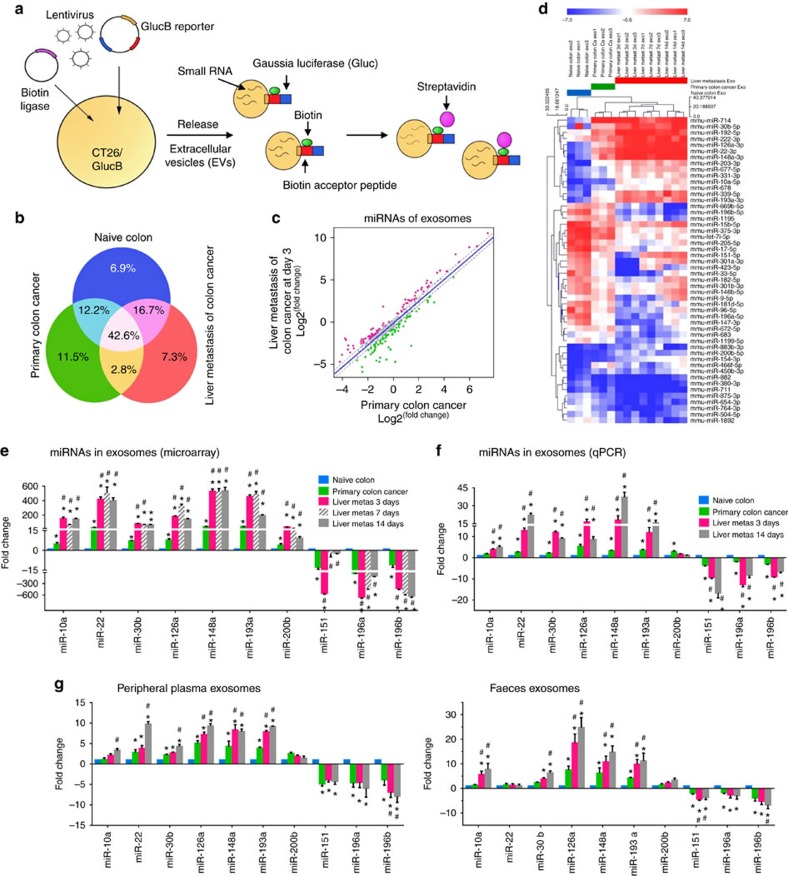
Identification of exosome miRNA profile that represents primary colon cancer and metastatic colon cancer in the liver. (**a**) Schematic diagram for isolation of extracellular vesicles (EVs) from colon cancer CT26 cell line with multimodal imaging report. CT26 cells stably transduced with a lentiviral vector expressing membrane-bound *Gaussia* luciferase (GlucB) and biotin ligase (BirA). (**b**) Venn diagram summarizing unique and shared exosomal miRNAs detected in the tissues of naïve colon, primary colon cancer and metastatic mouse colon cancer in the liver using miRNA microarray data (*n*=5 mice per group). (**c**) Microarray data visualization by scatter plot comparing exosomal miRNAs detected in primary colon cancer (*x* axis) and metastatic colon cancer in the liver at day 3 (*y* axis) after a CT26 cell intrasplenic injection. (**d**) Heat map depicting changes in miRNAs with a statistically significant (*P*<0.05) change in the exosomal miRNAs from normal mouse colon, primary colon cancer tissue and metastatic colon cancer in the liver at days 3, 7 and 14 after injection of CT26 cells (*n*=3 mice per group). All tumour-derived exosomes were isolated with streptavidin magnetic beads. Microarray analysis results (**e**) and qPCR verification (**f**) of selected exosomal miRNAs from the source as described in **d**. (**g**) qPCR analysis of the plasma- (left panel) or faeces- (right panel) derived exosomes from the source are depicted in **d**. *P<0.05 versus naïve colon; ^#^*P*<0.05 versus primary colon cancer (two-tailed *t*-test). Data are representative of three independent experiments (error bars, s.e.m.).

The presence of EV RNAs in tissues and in fluids, including blood, together with the changes in EV RNA expression in various pathological conditions, has implicated EV RNAs as informative biomarkers of progression and early diagnosis for cancer^17^,^18^,^19^,^20^,^21^,^22^. However, EVs are released from many different types of cells, including tumour and non-tumour cells. The challenge is to distinguish between EVs released from tumour cells and those released from non-tumour cells. To achieve this overall goal, we generated stably transduced colon cancer CT26 cells with lentivirus vectors^16^ that enable the isolation of EVs from CT26 tumour cells (Fig. 1a). The expression of this construct, as described in Fig. 1a, provides a biotin binding moiety for isolation of exosomes, as well as *Gaussia* luciferase (Gluc) and green fluorescent protein tags for monitoring CT-26 tumours in organs and biological fluids (Supplementary Fig. 1a). The results generated from luciferase assays with coelenterazine (CTZ) indicate that the EVs released from CT26 cells are stably transfected with the lentivirus vector expressing Gluc, biotin acceptor peptide and TM domain and that these cells exhibited a higher luciferase activity compared with the cells expressing Gluc without the TM domain (Supplementary Fig. 1b). Moreover, more Gluc activity in the medium and less Gluc activity in the whole cell lysates was detected in the cells transfected with membrane-bound Gluc than in cells transfected with GlucB. Lentiviral vector stable transfection had no significant effects on the numbers of exosomes that were shed or the levels of miR-193a, miR-18a or miR-126a in the exosomes released from stably transfected CT26 cells compared with wild-type CT26 cells (Supplementary Fig. 1c,d). To characterize the tumour EVs released from primary and metastatic cancer in the liver of colon cancer mice, CT26 cells stably expressing both EV-Gluc and biotin ligase BirA were administered to BALB/c mice by colonic submucosa or intrasplenic injection as described^23^. Two weeks after injection, a tumour was evident in hematoxylin and eosin (H&E)-stained sectioned colon and liver (Supplementary Fig. 2a) and in confocal imaging of sectioned liver (Supplementary Fig. 2b). Gluc and BirA expression were visualized with green fluorescent protein-tagged Gluc and red fluorescent mCherry-tagged BirA in metastatic lesions in the liver, but not in the adjacent normal liver tissue (Supplementary Fig. 2b). To estimate Gluc luciferase activity *in vivo*, CTZ or phosphate-buffered saline (PBS; control) was injected via tail vein injection into BALB/c mice-bearing CT26 tumours with Gluc expression. Five minutes after the injection, CTZ-injected mice revealed a significant amount of Gluc signal in the liver of GlucB-expressing mice, but not in PBS-injected tumour-bearing mice (Supplementary Fig. 2c). These results confirm an *in vivo* biological activity and stability of Gluc imaging reporter in liver metastatic CT26 tumour cells.

To further determine if tumour-specific exosomes can be isolated from metastatic liver, exosomes were isolated from liver metastasis of colon cancer, followed by purification with sucrose gradient centrifugation. Isolated exosomes were dot blotted on nitrocellulose membranes followed by probing with anti-CD63 antibody and streptavidin-conjugated Alexa Fluor 488. Dot blot analysis showed that exosomes from both naïve mouse liver and liver with metastatic colon cancer expressed exosomal marker CD63 with the same fluorescent intensity (Supplementary Fig. 3a). However, biotinylated EV-GlucB could be detected in liver with metastatic colon cancer-derived exosomes, but not in naïve mouse liver-derived exosomes (Supplementary Fig. 3a). Western blot analysis demonstrated the presence of exosomal protein marker CD63 and the absence of the endoplasmic reticulum protein Calnexin in exosomes (Supplementary Fig. 3b). Exosomal morphology and size distribution were further evaluated using electron microscopy (Supplementary Fig. 3c) and Zetasizer Nano ZS analysis (Supplementary Fig. 3d), respectively. Metastatic liver exosomes had a diameter of 48.6±4.6 nm (means±standard error of the mean (s.e.m.)) and were smaller than naïve mouse liver-derived exosomes, which had a diameter of 83.6±7.8 nm (means±s.e.m.) (Supplementary Fig. 3d, left). The number of exosomes released from liver metastasis of colon cancer was much higher than that from the liver of naïve mice (Supplementary Fig. 3e).

### Exosomal miRNA as an indicator for tumour progression

Identification of a unique miRNA profile encapsulated in tumour cell exosomes but not in non-tumour cell exosomes is essential for clinical applications. Tumour exosomal miRNA has recently been extensively studied for use as a diagnostic marker^20^,^24^,^25^,^26^,^27^,^28^,^29^,^30^,^31^,^32^,^33^ and as an indicator for disease progression^3^,^10^,^34^. To further determine whether our approach could identify an exosomal miRNA profile that mirrors disease progression, we performed an miRNA microarray and did comparative analysis of miRNome in exosomes isolated from normal colon, primary colon cancer tissue and colon tumour metastasis to the liver (Fig. 1b, Supplementary Fig. 2a). Among these differentially expressed miRNAs, 6.9% of the miRNA was uniquely detected in normal colon tissue-derived exosomes, 11.5% of the miRNA was detected in primary colon tumour exosomes and 7.3% of the miRNA was detected in metastatic tumour exosomes (Fig. 1b). Next, exosomal miRNA profiles from liver metastasis of colon cancer were compared with the exosomal miRNA profiles from primary colon tumour tissue. In the scatter plot (Fig. 1c), each point represents the expression value of a given miRNA. Compared with exosomal RNAs from primary colon tumour tissue, exosomal RNA profiles from liver metastasis of colon cancer displayed a different distribution (Fig. 1c). The green and red dots represent the higher level of exosomal miRNAs detected in the primary colon cancer and liver metastasis, respectively, and the grey dots represent similar levels of exosomal miRNAs detected in primary and metastatic colon cancer. The criteria used to screen differences in miRNA encapsulation in the exosomes between normal tissue versus primary colon tumour tissue, and primary colon tumour tissue versus metastatic tumour tissue were based on fold changes of >3.0 or <−3.0. Fifty of the miRNAs met these criteria and were selected for further analysis (Table 1). A heat map of the 50 selected miRNAs demonstrated a gene cluster and sample cluster according to the level of miRNAs in the exosomes from normal tissue, primary colon tumour tissue and metastatic colon cancer in the liver (Fig. 1d). To verify the microarray results, seven of the upregulated miRNAs and three of the downregulated miRNAs in liver metastasis of colon cancer (Fig. 1e) were randomly chosen and confirmed by qPCR (Fig. 1f). The qPCR results indicated that these miRNAs were encapsulated into exosomes that were subsequently released into circulation (Fig. 1g, left panel) and excreted into the gut (Fig. 1g, right panel) from metastatic CT26 tumour cells in the liver. The stable character of exosomal miRNAs in biological fluids and faeces indicates that miRNAs in exosomes can be used as potential biomarkers for clinical diagnosis and prognosis.

### Tumour suppressor miRNAs selectively sort into exosomes

**Figure 2:**
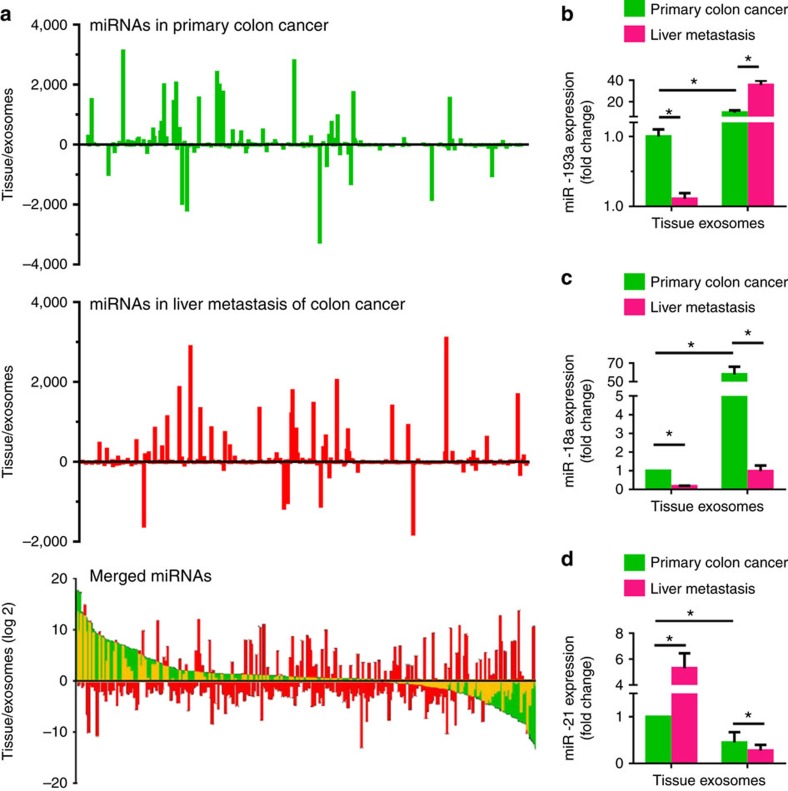
miRNA profile from exosomes is different from their donor cells. (**a**) Comparative analysis of the miRNome in exosomes and exosome donor tissues using a microarray. miRNAs from exosomes and exosome donor tissues, including primary colon cancer (top panel) and liver metastasis (middle panel), were quantitatively analysed and expressed as a ratio of miRNAs from exosome donor tissues/exosomes. The similarity of each individual miRNA distribution in primary colon cancer and liver metastasis was analysed by overlaying each and are shown in yellow (bottom panel). Expression of miR-193a (**b**), miR-18a (**c**) and miR-21 (**d**) in the exosomes and exosome donor tissues, including primary colon cancer and liver metastasis of colon cancer, were assessed by qPCR. *P<0.05 (two-tailed *t*-test). Data are representative of three independent experiments (error bars, s.e.m.).

To determine whether the miRNA repertoires of exosomes differ from those of their donor cells, the profiles of miRNAs from exosomes and their donor cells were quantitatively analysed. Scatter plot (Supplementary Fig. 4a,b) results demonstrated a difference in exosomal RNA profiles from primary colon tumour (Supplementary Fig. 4a) and naïve colon (Supplementary Fig. 4b) from their donor tissues. We then calculated the ratios of any given miRNA from exosomes and their donor cells (Fig. 2a). Our data suggest that loading miRNAs into exosomes is not a passive process (Fig. 2a). In primary colon tumour-derived exosomes, 26.7% of miRNAs analysed are higher and 47.5% of miRNAs are lower when compared to exosomal donor tumour cells; 25.8% of miRNAs analysed are present in the exosomes in both types of cells (Fig. 2a, top panel). However, when CT26 colon tumour cells metastasize to the liver, the pattern of the CT26 tumour exosomal miRNA profile is altered (Fig. 2a, middle panel) in comparison to the pattern of the exosomal miRNAs expressed in the primary colon tumour. Some of the exosomal miRNAs retain the same patterns as indicated in yellow (Fig. 2a, bottom panel) regardless of whether they are in the primary colon tumour or metastatic colon cancer in the liver.

To investigate whether the level of tumour exosomal miRNA is sorted based on the miRNA biological function, the miRNA profiles were summarized (Table 2) based on miRNA-oncogenic versus tumour-suppressive effects. We found that among the three types of exosomes isolated from normal colon tissue, primary colon tumour tissue and metastatic colon tumour in the liver, metastatic CT26 colon-derived exosomes have the highest level of tumour-suppressive miRNAs and the lowest level of oncogenic miRNAs. We then investigated whether such a difference is determined by the level of miRNA expressed in the parent tissue. As summarized in Table 2, we noticed that most of the miRNAs (miR-10a-5p, miR-193a-3p, miR-200b-5p, miR-222-3p) that are actively sorted into exosomes have tumour suppressive effects involving cell growth suppression, whereas miRNAs (miR-196a/b, miR-181d-5p, miR-155-5p) that have oncogenic effects are retained in the tumour cells even though the levels of the oncogenic miRNAs are higher in their donor cells than in the exosomes. This finding was further demonstrated by reverse transcription-quantitative PCR (RT-qPCR) analysis of tumour-suppressive miR-193a (Fig. 2b), miR-18a (Fig. 2c) and oncogenic miR-21 (Fig. 2d), as an example. Our results indicate that the level of miR-18a and miR-193a in the exosomes from either primary colon tumour tissue or metastatic liver of colon tumour is higher than in their donor tumour tissues. In contrast, the level of oncogenic miR-21 was much higher in the primary colon tumour tissue and metastatic colon tumour in the liver than in their exosomes. Collectively, these data suggest that oncogenic miRNAs are upregulated and that tumour suppressive miRNAs are downregulated in the tumour, and this phenomenon is clearly observed in metastatic colon tumour in the liver. Sorting oncogenic miRNAs from exosomal donor cells into their exosomes is suppressed, whereas sorting tumour suppressive miRNAs into exosomes is enhanced. This may be one of the mechanisms underlying tumour exosome-mediated promotion of tumour progression.

### Effects of microenvironment on the exosomal miRNA

**Figure 3:**
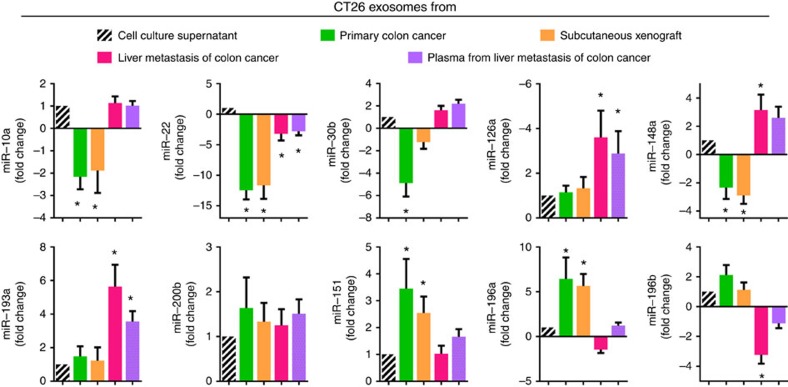
Microenvironment alters the composition of tumour exosome miRNA profiles. Exosomal miRNAs isolated from the source listed in this figure were quantitatively analysed by qPCR. *P<0.05 (two-tailed *t*-test). Data are representative of three independent experiments (error bars, s.e.m.).

The majority of the published data show the biological activities of tumour EVs using *in vitro c*ultured tumour cell-derived EVs. This may not accurately represent the case for tumour EV released from tumour tissue because multiple factors derived from tumour tissue have a remarkable effect on the composition of tumour EVs, and those factors do not exist in the culture medium. As proof of concept, we compared the levels of selected miRNAs (Fig. 3) present in exosomes released from *in vitro* cultured CT26 cells (culture medium environment) from primary colon cancer, CT26 cell subcutaneous xenograft, metastatic CT26 tumour isolated from mouse liver and from exosomes circulating in the peripheral blood. The results generated from qPCR show that miR-126a, miR-148a and miR-193a are significantly higher in the exosomes released from metastatic CT26 cells and circulating in the peripheral blood of metastatic colon cancer in the liver, but not from primary colon cancer or subcutaneous xenografts. However, miR-22, miR-196a and miR-196b are decreased in the exosomes from metastatic colon tumour in the liver (Fig. 3) compared with exosomes from *in vitro* cultured CT26 cells. These changes are specific as other miRNAs, including miR-10a, miR-30b, miR-200b and miR-151, are not changed in amount regardless of the origin, whether from the exosomes of cultured tumour cells or metastatic CT26 cells, suggesting that the microenvironment has an effect on the composition of the exosomal miRNA profile. To further confirm that exosomes isolated from colon tumour tissue do not contain other intracellular microvesicles such as multivesicular bodies, the exosomes were isolated from the supernatants of 12 h-*ex vivo*-cultured CT26 colon cancer cells isolated from colon tumours. Exosomal miRNAs isolated from the supernatants of *ex vivo*-cultured CT26 colon cancer cells and from CT26 colon cancer tissue were qPCR analysed. The results suggest that levels of the exosomal miRNAs in the exosomes from the supernatants of *ex vivo*-cultured CT26 cells are not significantly different from exosomal miRNAs in the CT26 colon cancer tissue (Supplementary Fig. 5).

### Oncosuppressor miR-193a directly targets *Caprin1*

**Figure 4:**
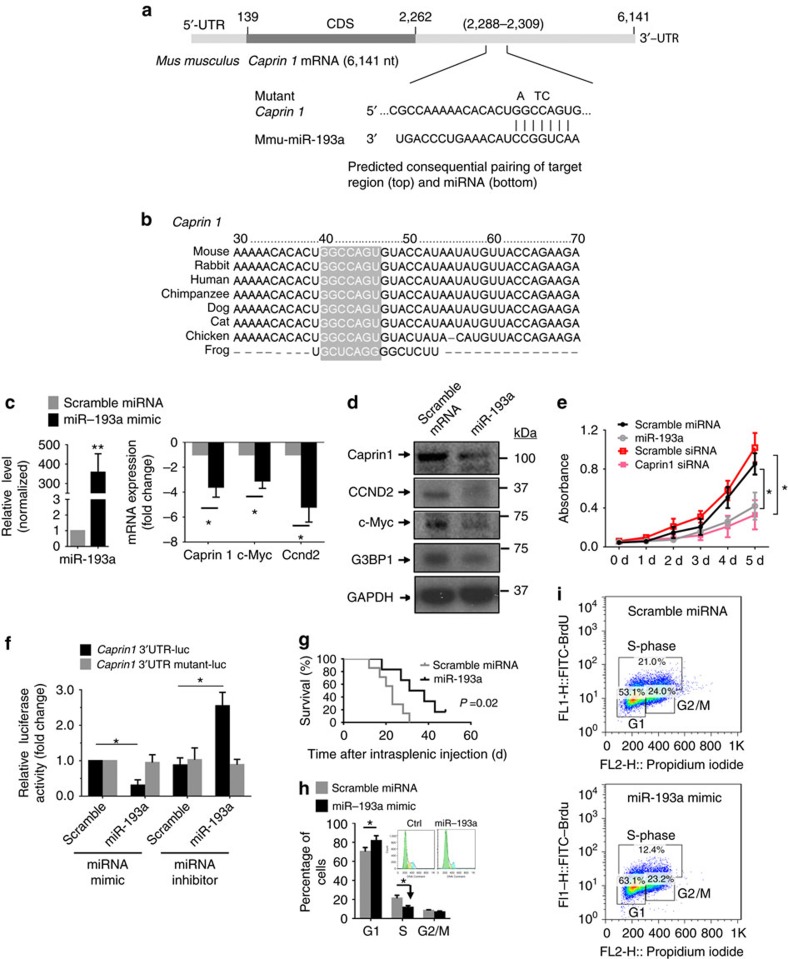
miR-193a suppresses the progression of CT26 colon cancer by directly targeting *Caprin1*. (**a**) Schematic diagram of the putative binding sites of miR-193a in the wild-type (WT) *Caprin1* 3′ untranslated regions (UTR). The miR-193a seed matches in the *Caprin1* 3′UTR are mutated at the positions as indicated. CDS, coding sequence. (**b**) Potential miR-193a binding sites on *Caprin1* (in grey) are broadly conserved among vertebrates. (**c**) Expression of miR-193a and candidate target gene *Caprin1* as well as downstream genes (Ccnd2, c-myc) in CT26 cells assessed by qPCR following transfection of miR-193a mimic and control scramble miRNA. (**d**) Expression of candidate miR-193a and candidate target genes Caprin1 as well as downstream genes (Ccnd2, c-Myc, G3bp1) in CT26 cells assessed by western blot, following transfection of miR-193a mimic and control miRNA for 72 h. (**e**) Proliferation of CT26 cells with miR-193a and potential target *Caprin1* knock down. Cell viability was detected from day 0 to 5 after transfection. (**f**) Luciferase activity assays of wild-type (WT) and mutated *Caprin1* 3′UTR luciferase reporters after co-transfection with miR-193a mimic, miRNA mimic control (scramble), anti-sense miR-193a or anti-sense negative control RNA in CT26 cells. The luciferase activity of each sample was normalized to the Renilla luciferase activity. The normalized luciferase activity of transfected control mimic miRNA was set as a relative luciferase activity of 1. (**g**) Survival of BALB/c mice after intrasplenic injection of CT26 cells with miR-193a overexpression (*n*=6 mice per group). (**h**) The cell cycle phase analysis of CT26 cells transfected with miR-193a mimic for 72 h using PI. The percentage of cells in the G1, S, and G2 phases are shown in the bar graph. (**i**) BrdU incorporation assay for cell cycle analysis of CT26 transfected with miR-193a mimic and control miRNA. Error bars represent s.e.m. *P<0.05 and **P<0.01 (two-tailed *t*-test). Each data point was measured in triplicate (error bars, s.e.m.).

We further hypothesize that exporting tumour-suppressive miRNA such as miR-193a from exosome donor cells into exosomes is a benefit for colon cancer metastasis to the liver. We first searched miRNA databases for potential miR-193a targets that may contribute or promote tumour progression. Three public miRNA databases (TargetScan, Pictar and MicroRNA) all predicted that cell cycle-associated protein *Caprin1* might be a target for miR-193a (Fig. 4a), and the 3′-UTR of Caprin1 contains a highly conserved binding site from position 2288 to 2309 for miR-193a (Fig. 4a,b). To determine whether miR-193a could target *Caprin1* in colon cancer cells, we transfected the mature mouse miR-193a mimic into CT26 cells. The CT26 cells overexpressing miR-193a (Fig. 4c, left panel) have significantly downregulated *Caprin1* mRNA expression (Fig. 4c, right panel) as well as *Caprin1* protein expression (Fig. 4d). We found that *CCND2* and *c-MYC*, which are regulated by *Caprin1*, are also decreased as a result of miR-193a treatment (Fig. 4c,d). The impact of miR-193a overexpression on the inhibition of cell proliferation was further confirmed by the *Caprin1* siRNA knockdown in CT26 colon cancer cells (Fig. 4e). To ascertain the direct effect of miR-193a on *Caprin1*, a mutant construct that would disrupt the predicted miR-193a binding site was generated from pEZX-MT01-Caprin1 containing a full length 3′UTR of Caprin1 mRNA (Gene Accession: NM_001111289). We performed a luciferase reporter assay by co-transfecting a vector containing *Caprin1* 3′UTR-fused luciferase and miR-193a or control miRNA as a negative control. Overexpression of miR-193a decreased the luciferase activity of the reporter with the 3′UTR of *Caprin1* by approximately 56% in CT26 cells (Fig. 4f). However, the mutation that disrupted the binding site for miR-193a entirely restored luciferase activity. Moreover, overexpression of anti-sense miR-193a (miRNA inhibitor) caused induction of luciferase; however, there was no inductive effect of the anti-sense miR-193a on the activity of the reporter with a mutant 3′UTR of *Caprin1* (Fig. 4f). These results demonstrate that *Caprin1* is a target of miR-193a in colon cancer cells. The tumour suppression role of miR-193a was further supported by the fact that overexpression of miR-193a inhibited CT26 cell proliferation and significantly prolonged survival of colon cancer-bearing mice (Fig. 4g). Cell cycle assessment suggested that miR-193a causes a G1 phase arrest in the cell cycle (Fig. 4h,i).

### MVP regulates the loading of miR-193a to exosomes

**Figure 5:**
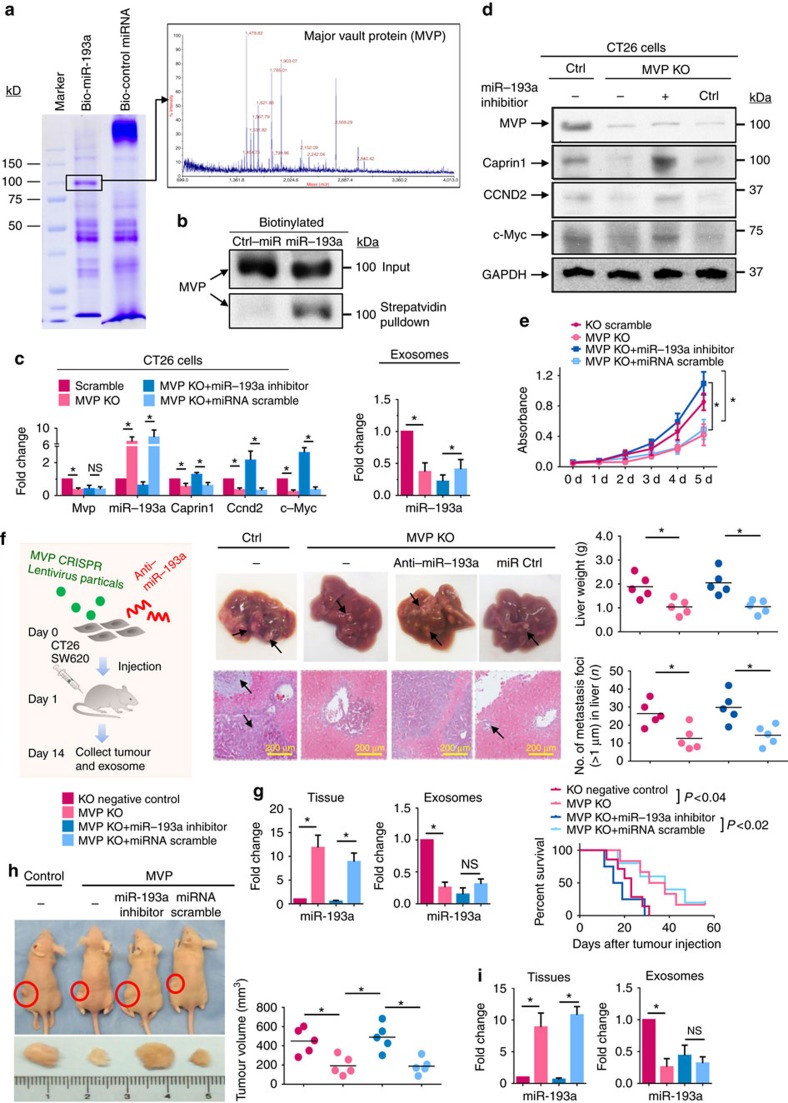
Sorting of miR-193a from cell to exosomes through major vault protein (MVP). (**a**) Biotin-miR-193a complex was pulled down from whole cell extracts using streptavidin beads and then analysed by electrophoresis followed by Coomassie blue staining (left panel). MALDI-TOF analysis of tryptic peptides (right panel) from the band indicated (left panel). (**b**) Western blot analysis expression of MVP proteins from before (top panel) and after streptavidin pulldown (bottom panel) of lysates of CT26 cells transfected with Bio-miR-193a or control miRNA. (**c**) MVP knockout (KO) CT26 cells were generated using the CRISPR/Cas9 system. qPCR-quantification of mature miR-193a, MVP, Caprin1, CyclinD and c-MYC expressed in CT26 cells (left panel) and CT26 exosomes (right panel) after the cells were treated as indicated. (**d**) Western blot analysis showing the level of MVP, Caprin1, CCND2 and c-MYC in cell lysates treated as indicated. (**e**) Proliferation of MVP KO CT26 cells treated as indicated. Cell viability was detected from day 0 to 5 after transfection. (**f**) Schematic representation (left panel) of treatment schedule as indicated. Representative livers (middle top panel) (metastatic nodules shown by arrows) and H&E-stained sections of livers (middle bottom panel, × 400 magnification, scale bar 200 μm) from tumour-bearing BALB/c mice (*n*=5 per group). Liver weight (right, top panel) and number of metastatic foci in liver (right bottom panel) were quantitatively analysed. (**g**) Mature miR-193a in tumour tissue (left panel) and tumour exosomes (middle panel) was quantified by qPCR. Survival analysis of BALB/c mice after intrasplenic injection of CT26 cells treated as indicated (right panel) (*n*=9 per group). (**h**) Representative images of xenografts in SW620 tumour-bearing nude mice (left panel) (*n*=5 mice per group). Changes of tumour volumes in an SW620 xenograft model (right panel). Liver tumour volume was used to evaluate tumour size using the following formula: nodule volume=(width)^2^ × length/2. (**i**) qPCR-quantification of mature miR-193a in exosomes and tissues of tumour in SW620 xenograft mice. *P<0.05 (two-tailed *t*-test); NS represents non-significance. Each data point was measured in triplicate (error bars, s.e.m.).

Although the miRNA repertoires of exosomes differ from those of their donor cells, the explanation or mechanism for how this occurs is still unknown. We hypothesized that host factor(s) might play a role in miRNA sorting from exosomal donor cells to their exosomes. To test our hypothesis, biotin-labelled miR-193a complex was isolated from exosomal lysates using streptavidin beads. A typical staining pattern of the Bio-miR-193a complex obtained from CT26 exosomal extracts on sodium dodecyl sulfate polyacrylamide gel electrophoresis (SDS-PAGE) is shown in Fig. 5a, left panel. In-gel digestion-MALDI-TOF mass spectrometry (MS) analysis was carried out for identification of proteins that are specifically present in the Bio-miR-193a complex sample but not in the control bio-miRNA complex. Major vault protein (MVP) was subsequently identified as a potential miR-193a binding protein by MS (Fig. 5a, right panel), and this interaction was verified by western blot (Fig. 5b, Supplementary Fig. 6a). CT26 cells transfected with MVP knock out (KO) MVP sgRNA CRISPR Lentivirus (>1 × 10^7^ IU ml^−1^) have low levels of MVP detected (Fig. 5c,d) but the level of miR-193a is increased in the cells (Fig. 5c, left panel). This finding is inversely correlated with the levels of miR-193a in the exosomes (Fig. 5c, right panel). The accumulation of miRNA in the MVP KO CT26 cells is miR-193a-specific since no change of miR-126a was observed due to MVP KO (Supplementary Fig. 6b). Collectively, these data suggest that miR-193a sorting into exosomes is MVP-dependent. Next, to determine whether MVP interacts with miR-193a, immunoprecipitation of MVP was carried out. The qPCR results indicate that MVP interacts with miR-193a in an MVP immunocomplex dose-dependent manner (Supplementary Fig. 7a). The results generated from MVP gene overexpression in CT-26 cells suggest that the higher the level of MVP, the lower the level of miR-193a (Supplementary Fig. 7b,c). The higher level of MVP had similar results as higher levels of Caprin1 (Supplementary Fig. 7d). To further understand the effects of MVP KO on the cells, we compared cell proliferation of MVP KO CT26 cells with scramble KO control cells. MVP KO led to the repression of tumour cell proliferation (Fig. 5e). This result agreed with the finding that cyclin D2 (CCND2) and c-MYC are decreased at both transcriptional (Fig. 5c, left panel) and protein levels (Fig. 5d). Inhibition of miR-193a expression reversed the effects of MVP KO on repressive expression of Caprin1, CCND2 and c-MYC (Fig. 5d), eventually leading to induction of cell growth (Fig. 5e). The results generated from a mouse colon cancer model with liver metastasis further supports that miR-193a exported via exosomes by MVP promotes tumour progression (Fig. 5f). MVP KO in CT26 colon tumour cells resulted in the inhibition of liver metastasis of colon cancer (Fig. 5f, middle and right panels), and this result correlates with an increase in miR-193a in CT26 cells and a decrease in miR-193a in exosomes (Fig. 5g, left panel). Knock down of miR-193a expression by miR-193a inhibitor enhanced tumour metastasis in liver and decreased survival of colon cancer-bearing mice (Fig. 5g, right panel).

The MVP-mediated promotion of tumour progression through miR-193a is also demonstrated by subcutaneous injection of human colon cancer SW620 cells into nude mice (Fig. 5h). Mice injected subcutaneously with SW620 cells showed remarkable tumour growth over 14 days. However, mice injected subcutaneously with MVP KO SW620 cells have a much slower tumour growth rate. This repression on tumour growth was partially reversed by knocking down the expression of miR-193a (Fig. 5h,i). Analysis of miR-193a levels in tumours by qPCR indicated that MVP KO caused the accumulation of miR-193a in cells (Fig. 5i, left panel), with a concomitant decrease of miR-193a in the exosomes (Fig. 5i, right panel). Collectively, these data suggest that MVP regulates miR-193a sorting into exosomes and that the accumulation of miR-193a in the exosomal donor cells as a result of KO MVP is detrimental to tumour cells. By contrast, reduction of miR-193a by MVP-dependent sorting into exosomes leads to tumour cell proliferation and to a faster cell cycle, eventually enhancing tumour cell growth and metastasis.

### Higher levels of exosomal miR-193a lead to more aggressive disease

**Figure 6:**
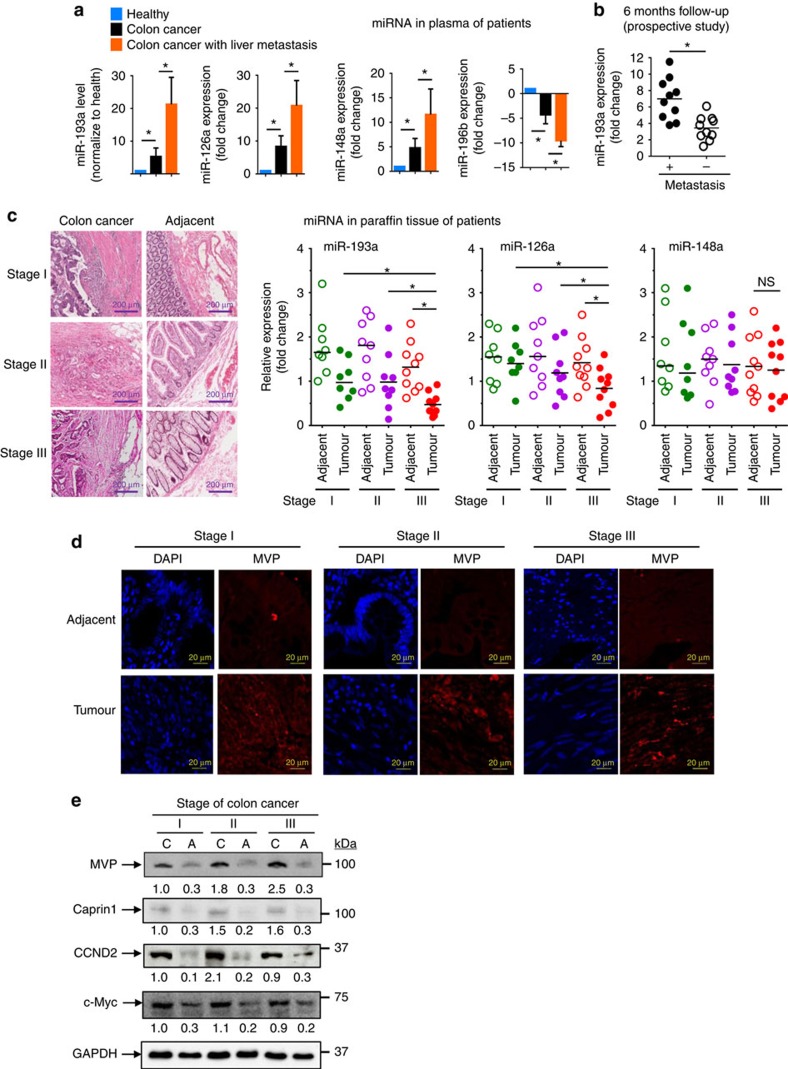
Induction of exosome miR-193a in peripheral blood increases the risk of metastatic colon cancer in the liver of patients. (**a**) qPCR-quantification of mature miR-193a, miR-126a, miR-148a and miR-196b in exosomes of plasma collected from colon cancer patients with (*n*=15) or without (*n*=25) liver metastasis. (**b**) qPCR analysis of miR-193a level in the exosomes from peripheral blood of colon cancer patients without metastasis and follow-up investigation carried out six months after diagnosis. (**c**) Representative H&E-stained sections of colon tumour tissue (× 200 magnification) from patients at various cancer stages. Scale bars, 200 μm (left panel). qPCR analysis of miR-193a, miR-126a and miR-148a expression in colon cancer tissue and adjacent non-tumour tissue from the same patients (right panel). (**d**) Representative MVP (red) expression in patient tumour sections with Alexa Fluor 594 dye labelling anti-MVP antibodies (red) visualized with confocal microscopy. (**e**) Western blot analysis showing the level of MVP, Caprin1, Cyclin D and c-MYC in colon cancer tissue (**c**) and adjacent non-tumour tissue (**a**) from the same patient at various stages (I, II and III) as indicated. GAPDH used as a loading control. Data are representative of three independent experiments. *P<0.05 and **P<0.01 (two-tailed *t*-test; error bars, s.e.m.); NS represents non-significance.

To further determine whether our finding as described above can be translated into a clinical application, three upregulated (miR-193a, miR-126 and miR-148a) and one downregulated miRNA (miR-196b) found in the exosomes isolated from metastatic liver in a mouse colon cancer were analysed in samples from colon cancer patients by qPCR. Twenty-five colon cancer patients without metastasis and 15 colon cancer patients with liver metastasis were enrolled. Overall, the exosomes isolated from the peripheral plasma of colon cancer patients exhibited higher levels of miR-193a, miR-126 and miR-148a and lower levels of miR-196b compared to healthy controls (Fig. 6a). Furthermore, the exosomes isolated from the plasma of colon cancer patients with liver metastasis displayed higher levels of miR-193a, miR-126 and miR-148a and lower levels of miR-196b compared to the plasma of colon cancer patients having no metastasis (Fig. 6a). The liver metastasis incidence was further investigated with a 6-month follow-up after primary diagnosis. This prospective study indicated that colon cancer patients with high levels of miR-193a in exosomes from peripheral blood have a higher risk of metastasis (Fig. 6b). Colon cancer stages of patients were classified by H&E staining with an estimate of the levels of infiltration of a particular cancer (Fig. 6c, left panel). The miRNAs expressed in tumour tissue and adjacent normal tissue (used as a control) were analysed by qPCR. The level of miR-193a in the tumour was lower than in adjacent tissue in all three stages of colon cancer. The downregulation of miR-193a in tumour tissue was stage-dependent (Fig. 6c, second panel from left). MiR-126a downregulation only appeared in stage III of colon cancer when compared to adjacent normal tissue (Fig. 6c, third panel from left). Although miR-148a increased in the plasma of colon cancer patients, no difference in miR-148a level between tumour tissue and adjacent tissue was evident (Fig. 6c, right panel). Induction of MVP in colon cancer tissue is stage-dependent and supported by confocal immunostaining (Fig. 6d) and immunoblotting results (Fig. 6e). Along with the increase of MVP, the induction of Caprin1, CCND2 and c-MYC was detected at the protein level (Fig. 6e).

## Discussion

Cancer EVs including exosomes play a role in promoting tumour progression. Most published data show the biological activities of tumour-derived EVs using cultured tumour cell-derived EVs, which may not accurately reflect the case for tumour cell-derived EVs released from tumour tissue. A major challenge for studying the biological activities of tumour cell-derived EVs released from tumour tissue is to develop methodology to isolate tumour-specific EVs from mixed EVs (refs 5, 9, 35, 36) and to validate the data generated from tumour tissue exosomes with a standard method such as *ex vivo* short-term cultured tumour cells isolated from the same tumour tissue as we described in this study. In this study, using a biotin-streptavidin-based detection approach, we present a methodology that could be suitable for the isolation of tumour cell-derived EVs from tumour tissue; however, it is not possible, at the moment, to distinguish between intracellular and extracellular origin of the isolated extravesicles. The fact that qPCR results generated with the tumour tissue-derived exosomal miRNA agreed with the data from exosomal miRNA isolated from peripheral blood of CT26 tumour-bearing mice but not naïve mice further warrants using this methodology for future research in the EV field. Moreover, the fact that colon tumour tissue-derived exosomal miRNA profile mirrors disease progression and is detected in the circulating exosomes further support this approach to identify new biomarkers of progression and early diagnosis for cancer. Therefore, this approach could provide an avenue for the isolation, characterization and functional analysis of any subpopulation of tissue-specific EVs. For example, circulating exosomes can be pulled-down with an antibody that recognizes a gut epithelial-specific marker, such as A33. Since higher levels of exosomal miR-193a in the peripheral blood of colon cancer patients is correlated with more aggressive disease, the level of circulating miR-193a in A33^+^ exosomes can be quantitatively analysed with qPCR. Therefore, exosomal miR-193a may be used as a biomarker for predicting progression of colon cancer.

However, with this methodology, we cannot exclude the possibility of isolating tumour exosomes that are contaminated with other intracellular vesicles. To further determine whether the vesicles isolated from tumour tissue represent tumour cell-derived exosomes without contamination with other intracellular microvesicles, exosomes were isolated from the supernatants of 12-h *ex vivo*-cultured CT26 cells isolated from colon tumour tissue. The results indicate similar miRNA profiles from the sucrose-purified exosomes isolated from either the supernatants of *ex vivo* 12 h-cultured CT26 cells or colon tumour, suggesting that intracellular microvesicle contamination in colon tumour-derived exosomes is negligible. In addition, based on the data published, other intracellular microvesicles, such as multivesicular bodies, have different properties than exosomes^35^,^37^. Collectively, our *ex vivo* approach for validation of the purity of tumour tissue-derived exosomes could be a criterion for measuring the purity of EVs isolated from tissues in general.

More importantly, in this study, our approach led to two new findings: (1) tumour suppressor miRNAs are sorted into tumour exosomes, whereas oncogenic miRNAs remain in the tumour cells; and (2) in severe disease, higher levels of tumour suppressor miRNAs are present in the tumour exosomes. These conclusions are also supported by the data generated from exosomes isolated from the peripheral blood of colon cancer patients. Therefore, these findings provide a solid foundation for further investigating the molecular mechanism(s) underlying how tumour cells selectively sort tumour suppressor miRNA out of cells. As proof of concept, we used tumour suppressor miR-193a to demonstrate this phenomenon. Our findings on the mechanism of sorting tumour suppressor miR-193a into exosomes have implications for other known tumour suppressor miRNAs, such as those listed in Table 2. Our findings also support future studies to determine whether tumour suppressor miRNAs have unique signal(s) recognized by the cellular complex. The activity of cellular machinery for sorting tumour suppressor miRNA into exosomes may cross-talk/communicate with a family of oncogenic factors, such as MVP, which we identified in this study. An improved understanding of the signals that regulate tumour suppression versus oncogenic miRNA sorting in/out of exosomes may provide new therapeutic strategies for the treatment and/or prevention of cancer that is due to dysregulation of cellular miRNA homeostasis.

**Figure 7:**
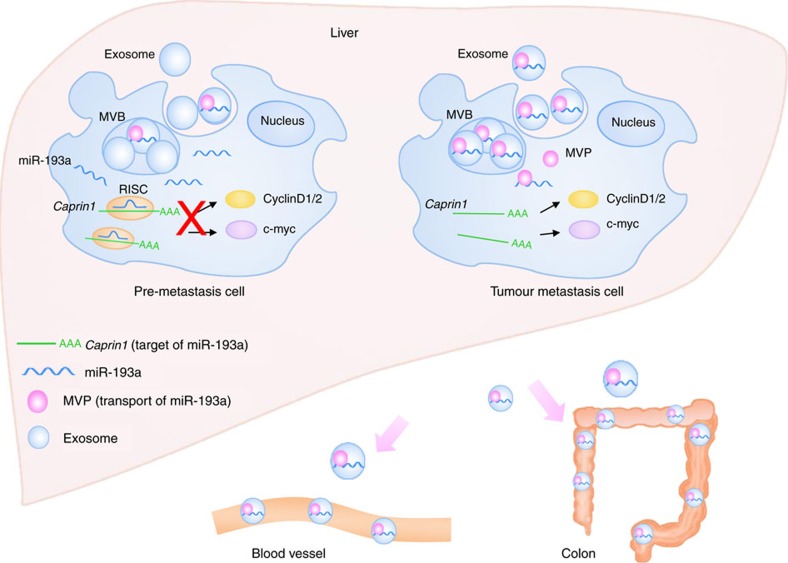
Proposed model for the mechanism of colon cancer metastasis to the liver involves exporting miR-193a via exosomes sorted by MVP. Abbreviations: MVB, multivesicular bodies; RISC, RNA-induced silencing complex.

MVP is overexpressed in multidrug-resistant cancer cells^38^,^39^,^40^,^41^,^42^,^43^,^44^,^45^. In this study, we demonstrated that MVP transports miR-193a from tumour cells to exosomes. As summarized in Fig. 7, MVP binds to tumour suppressor miR-193a, forming an MVP protein-miR-193a complex. Subsequently, this complex is packed into exosomes leading to the reduction of cytoplasmic miR-193a. The fact that MVP knockout causes miR-193a accumulation in cells instead of exosomes further supports this finding. Our study demonstrates that accumulation of miR-193a in the tumour cell leads to inhibition of tumour growth. The data presented in this study indicate that miR-193a targets the 3′UTR of Caprin-1 mRNA, leading to inhibition of production of caprin-1 protein. Caprin-1 is known to regulate the cell cycle and cell proliferation^46^,^47^,^48^. Caprin-1 directly binds to mRNAs for G3BP1 protein^46^,^48^, c-MYC (ref. 47) and cyclin D2 through its carboxy-terminal RGG-rich region. We found that higher levels of miR-193a in tumour cells cause cell cycle G1 arrest and cell proliferation repression through reduction of caprin-1 expression. The impact of miR-193a-mediated interruption of the caprin-1/G3BP-1/c-MYC/Cyclin D2 complex could be a potential target for anti-cancer therapeutic applications. However, we demonstrated that knockout of MVP in CT26 cells leads to miR-193a accumulation in the cells without affecting the levels of miR-126a or miR-148a, suggesting that MVP selectively targets miR-193a. We cannot exclude the possibility that MVP may target other miRNAs, in particular other tumour suppressor miRNAs, subsequently regulating sorting of these tumour suppressor miRNAs into exosomes. This result raises many questions to be addressed in future studies.

In addition to miR-193a targeting of caprin-1, other potential molecules could be targeted by miR-193a, such as GTPase Rab27b, which regulates exosome biogenesis^49^. Accumulation of miR-193a in tumour cells may also inhibit the release of exosomes via miR-193a-3p-mediated targeting of GTPase Rab27b, which has been reported by other groups^50^. Therefore, it is speculated that accumulation of miR-193a in tumour cells by knockdown of MVP may prevent exosome release, thus contributing to inhibition of tumour progression as well. It is well-known that the tumour microenvironment has dramatic effects on the outcome of tumour growth. Whether the tumour microenvironment affects the composition of the tumour exosome miRNA profile has not been fully investigated. In this study, we show that the levels of tumour exosomal miRNAs from metastatic colon cancer in liver is different from the profile generated from an orthotopic cecum tumour model, subcutaneous xenograft tumour model or *in vitro* cell culture-derived miRNA profile. This result implies that these significantly high levels of miRNAs present in the exosomes released from metastatic colon cancer in liver are potential candidates for prognosis and diagnosis of colon cancer, in particular for liver metastasis. The process of metastasis of colon cancer to liver depends on multiple interactions between cancer cells in the tumour and host-derived cells in the microenvironment in both the primary tumour and secondary organ; however, they often occur undetected in the patient. Thus, despite its devastating impact, metastasis continues to be diagnosed in its final stage when little can be done. Identifying the exosomal miRNAs representing liver metastasis could lead to the development of more accurate methods of early detection and intervention in the metastatic process.

Presently there is no perfect technology to isolate tissue-derived EVs; therefore, developing the technology we described is essential for studying the *in vivo* roles of EVs in general. Like any other technology further optimization is required to validate its usefulness. In addition developing a device or method to collect biofluid from tumour tissue in live animals is necessary to monitor tumour progression.

## Methods

### Cell culture

The BALB/c syngeneic colon carcinoma CT26 cell line, human colonic epithelial SW620 cell line and human embryonic kidney 293 cells (American Type Culture Collection, Rockville, MD, USA) were grown at 37 °C in 5% CO_2_ in Dulbecco's modified Eagle's medium (DMEM, Life technology) supplemented with 10% heat-inactivated EV-depleted fetal bovine serum (FBS), 100 U ml^−1^ penicillin and 100 μg ml^−1^ streptomycin.

Monthly, potential mycoplasma contamination of the cell lines used for this study was examined with the Universal Mycoplasma Detection Kit according to the protocol provided (Catalog Number 30-1012K, ATCC, Manassas, VA, USA). The primers used are derived from a conserved region within the 16S rRNA coding region in the mycoplasma genome. DNA originating from other sources, such as tissue samples or *E. coli*, is not amplified. A touchdown PCR regimen increases sensitivity of the assay and also enhances specificity. The following controls were used for the PCR method: negative control sample, a sample of sterile water or growth medium, which must produce no PCR signal; positive control sample, a sample from a standard mycoplasma organism, which must produce a positive PCR signal.

### Generation of a CT26 cell line expressing EV-GlucB and sshBirA

Stable HEK293T-packaged cells expressing Gluc or GlucB with sshBirA were generated by transduction with lentivirus expression plasmids (kindly provided by Dr Xandra O. Breakefield)^16^. All plasmids were transfected with lentivirus packing vectors pCMVdelta8.2 and VSV-G using the FuGENE reagent (Promega, WI, USA). Pseudovirus-containing culture medium was collected after 72 h of transfection, and the viral titre was estimated. CT26 cells (2 × 10^5^) in a six-well plate received 10 μg ml^−1^ of polybrene as well as an appropriate amount of viral stock in the medium. After selection by puromycin, the cells with the highest expression of EV-GlucB and sshBirA were sorted using a BD FACSAria III cell sorter (BD Biosciences, San Jose, CA, USA). Details of other methods used in this study are described in the Supplementary Experimental Procedures, and the results were confirmed by confocal fluorescence microscopy (Nikon, Melville, NY, USA).

### Isolation and purification of exosomes

To isolate exosomes from liver tissue, a 20-G catheter was inserted into the portal vein of anaesthetized mice. The inferior vena cava was cut to allow the pre-warmed (37 °C) perfusion buffer (Ca^2+^-Mg^2+^-free HBSS containing 0.5 mM EGTA, 10 mM HEPES and 4.2 mM NaHCO_3_; pH 7.2) to flow freely through the liver. The liver was perfused 7–10 ml min^−1^ until there was no evidence of blood in the perfusion medium. The liver was then perfused for 5 min with dissociation buffer (HBSS containing 10 mM HEPES and 4.2 mM NaHCO_3_ supplemented with Type I collagenase (0.05%) and trypsin inhibitor (50 μg ml^−1^); pH 7.5) pre-warmed to 37 °C. The perfused liver and xenograft from subcutaneous and submucosa colon cancer tissue as well as naïve colon tissue were removed and gently disaggregated with tweezers in dissociation buffer, followed by incubation at 37 °C for 1 h. The disaggregated samples were centrifuged at 1,000*g* for 10 min, 2,000*g* for 20 min, 4,000*g* for 30 min and 10,000*g* for 1 h with the supernatant being retained each time. The tissue exosomes were collected by centrifuging the samples at 100,000*g* for 1.5 h at 4 °C, and the pellet was suspended in ice-cold PBS. The PBS suspended pellet was further loaded on a sucrose gradient (8, 30, 45 and 60% sucrose in 20 mM Hepes, 20 mM Tris-Cl, pH 7.2) for purification of exosomes using a protocol as described^2^. Sucrose gradient-purified exosomes were then used for RNA isolation.

Exosomes from serum and faeces were isolated using the exoEasy Maxi Kit (Qiagen, Frederick, MD, USA) according to the manufacturer's instructions. Briefly, 1 volume of Buffer XBP was added to 1 volume of pre-filtered serum or faeces suspended in PBS using Millex-AA (Millipore, Billerica, MA, USA) and then mixed well. The sample/Buffer XBP mix was then added onto the exoEasy spin column. After they were washed with 10 ml Buffer XWP, the exosomes were eluted using 400 μl Buffer XE.

To isolate exosomes from tissue culture medium, 1 × 10^6^ of CT26 cells or CT26 cells isolated from collagenase-digested colon tumour tissue as described above were grown in 10 ml of DMEM (Life Technology) supplemented with 10% heat-inactivated EV-depleted FBS, 100 U ml^−1^ penicillin and 100 μg ml^−1^ streptomycin at 37 °C in 5% CO_2_ for 48 h or 12 h, respectively. The medium was collected and centrifuged at 1,000*g* for 10 min, 2,000*g* for 20 min, 4,000*g* for 30 min and 10,000*g* for 1 h, with the supernatant being retained each time. The exosomes were collected by centrifuging the samples at 100,000*g* for at least 2 h at 4 °C. The exosomes were further purified on a sucrose gradient (8, 30, 45 and 60% sucrose in 20 mM Hepes, 20 mM Tris-Cl, pH 7.2) using a protocol as described^2^.

Size distribution and concentration of exosomes were analysed at a flow rate of 0.03 ml per min using a Zetasizer Nano ZS (Malvern Instrument, UK) and NanoSight NS300 (Westborough, MA, USA), respectively.

### Isolation of biotinylated exosomes

CT26 cells stably co-expressing GlucB with sshBirA allows CT26-derived exosomes to display a membrane reporter, termed EV-GlucB. The reporter consists of Gluc fused to a biotin acceptor domain, which is metabolically biotinylated when expressed in CT26 cells in the presence of biotin ligase (ssBirA). Therefore, exosomes isolated from CT26 cells stably co-expressing GlucB with sshBirA are biotinylated and can be isolated from mixed EVs with streptavidin-coated beads. In brief, for the streptavidin-coated bead pull down assay, isolated exosomes (1 mg) were resuspended in 90 μl of reaction buffer (2 mM EDTA/PBS) and incubated with 10 μl of streptavidin MicroBeads (Miltenyi Biotec, Germany) in 500 μl of reaction buffer at 4 °C for 2 h. After three washes with separation buffer (2 mM EDTA and 0.5% bovine serum albumin (BSA)/PBS), exosomes pulled down with streptavidin-coated beads were ready for protein extraction or RNA isolation.

### Mouse model study

Eight- to 12-week-old female BALB/c mice and athymic immunodeficient nude mice were purchased from the Jackson Laboratory (Bar Harbor, ME, USA) and housed under specific pathogen-free conditions. Animal care was performed following the Institute for Laboratory Animal Research (ILAR) guidelines, and all animal experiments were done in accordance with protocols approved by the University of Louisville Institutional Animal Care and Use Committee (Louisville, KY, USA). The mice were acclimated for at least 1 week before any experiments were conducted and were randomly assigned to the treatment groups.

For animal models of metastatic colon cancer in the liver, 8- to 12-week-old female BALB/c mice (*n*=5 per group) were anaesthetized with a mixture of ketamine and xylazine by intraperitoneal injection and 1 × 10^6^ CT26 colon cancer cells were administered via intrasplenic injection following a left subcostal incision through the skin and the peritoneum to expose the spleen^23^. For the animal xenograft model, 8- to 12-week-old female nude mice (*n*=5 per group) were inoculated subcutaneously on both flanks with human colonic epithelial SW620 cells (1 × 10^6^). On day 14, mice were killed, and livers or tumours were removed for examination. Xenograft volumes were evaluated by caliper measurements of two perpendicular diameters and calculated individually using the formula: Volume=*a* × *b*^2^/2 (*a* represents length and *b* represents width). Xenograft samples were collected for engraftment, histologic evaluation (paraffin section) or exosome isolation. The blood samples were obtained by cardiac puncture and were fractionated by centrifugation; the plasma was stored at −80 °C until ready for use.

For an animal model of primary colon cancer, 1 × 10^5^ CT26 colon cancer cells were injected into the colonic submucosa of 8- to 12-week-old female BALB/c mice (*n*=5) via an endoscopic work station (Karl Storz—Endoskope, Tuttlingen, German). The mice were killed, and colon tumours were removed for study on days 14–21 post-injection.

### Clinical samples

All clinical samples, including tissue and serum samples, were collected in the Department of Surgery, Huai'an First People's Hospital, Huai'an, Jiangsu, China with written informed consent from all human participants. Approval for the study was granted by the Institute Research Ethics Committee at the Health Department of Huai'an. Stages of colon cancer reflect whether and how far the cancer had spread according to the criteria from the National Cancer Institute (http://www.cancer.gov/types/colorectal/patient/colon-treatment-pdq#section/_112).

### qPCR analysis of miRNA and mRNA expression

Total RNA was isolated from cells, exosomes and tissue using a miRNeasy mini kit (Qiagen) and from serum using the exoRNeasy Serum/Plasma Midi Kit (Qiagen). For the isolation of RNA from paraffin-embedded tissues, four sections at 5 μm were placed in a tube, deparaffinized using xylene (Fisher) and rehydrated with decreasing concentrations of ethanol and PBS. Total RNA was isolated with the miRNeasy mini kit (Qiagen). The quantity of mature miRNAs expressed was determined by quantitative real-time PCR (qPCR) using a miScript II RT kit (Qiagen) and miScript SYBR Green PCR Kit (Qiagen) with Qiagen predesigned primers. All kits were used according to the manufacturer's instructions. A U6 transcript was used as an internal control to normalize RNA input. For analysis of MVP, Caprin1, c-myc and cyclin D2 mRNA expression, 1 μg of total RNA was reverse transcribed using SuperScript III reverse transcriptase (Invitrogen), and quantitation was performed using primers (Eurofins) with SsoAdvancedTM Universal SYBR Green Supermix (BioRad). GAPDH was used for normalization. The primer sequences are listed in Supplementary Table 1. qPCR was run using the BioRad CFX96 qPCR System with each reaction run in triplicate. Analysis and fold change were determined using the comparative threshold cycle (Ct) method^51^. The change in miRNA or mRNA expression was calculated as fold change.

### Analysis of miRNA microarray

miRNA expression profiling including exosomes and their donor cells or tissues was performed using the Qiagen miScript miRNA PCR Array Mouse miRBase Profiler (Cat# 331223) using an Applied Biosystems ViiA 7 Real-Time PCR System. Normalization to endogenous control genes included SNORD61, SNORD68, SNORD72, SNORD95 and RNU6 to correct for potential RNA input or RT efficiency biases. miRNA data generated from the tissue and EVs were comparatively analysed by the online free data analysis software at http://pcrdataanalysis.sabiosciences.com/mirna. Quantile normalization and subsequent data processing were performed using software R. Heat maps and scatter plots representing differentially regulated genes were generated using software R. Ingenuity pathway analysis was used to generate a network for predicting target genes in exosomes with high or low levels of miRNAs in liver metastasis of colon cancer.

### Site-directed mutagenesis within the *Caprin1* promoter

We utilized two algorithms that predict the mRNA targets of miRNAs, TargetScan (http://www.targetscan.org) and microRNA (http://www.microRNA.org), and Pictar (http://http://pictar.mdc-berlin.de/). *Caprin1* was selected by both online tools for having highly conserved 3′ untranslated region (3′ UTR) sites. To determine the ability of miR-193a to target the 3′ UTR-*Caprin1* activity, a luciferase reporter containing 3,243 bp of the *Caprin1* 3′ UTR in the pEZX-MT01 vector was purchased from GeneCopoeia (Cat^#^: MmiT072744-MT06, Rockville, MD, USA). The mutant of *Caprin1* 3′ UTR was generated with the oligonucleotide primer *Caprin1*-Mut, which was designed to specifically disrupt putative *Caprin1* at its 3′ UTR site. Q5 Site-Directed Mutagenesis Kit (New England Biolabs, MA, USA) was used in conjunction with specific primers (Supplementary Table 1) to introduce *Caprin1* 3′ UTR mutations in the pEZX-MT01 construct according to the manufacturer's instructions. After mutant strand synthesis and ligation, resultant plasmids were introduced into *E. coli*, and transformants were selected using kanamycin resistance. The DNA sequence of the mutants was confirmed by DNA sequencing.

### Transient transfection and luciferase reporter assay

Murine colon cancer CT26 cells were plated in 24-well plates at a density of 3.0 × 10^4^ cells per well in antibiotic free RPMI-1640 medium supplemented with 10% FBS. *Caprin1*-pEZX-MT01 (100 ng) or mutant luciferase reporter (100 ng) were transfected using FuGENE HD Transfection Reagent (Roche Applied Science, Indianapolis, IN, USA) with 10 pmol of mimic mmu-miR-193a and Opti-MEM Reduced Serum Medium (Invitrogen, Carlsbad, CA, USA). For all reporter assays, the cells were harvested 48 h post-transfection using Promega's Passive Lysis buffer. The activities of luciferase in cell lysates were determined using the Dual-Luciferase Reporter Assay System (Promega). Relative expression (fold-change) was determined by dividing the averaged normalized values from mock transfection. Values were averaged as indicated in the figure legends.

### Protein identification by MALDI-TOF mass spectrometry

Protein bands were excised from 10% SDS-PAGE gel stained with colloidal Coomassie blue (Bio-Rad, Hercules, CA, USA) and were incubated in 50 mmol l^−1^ NH_4_HCO_3_/50% acetonitrile at 22 °C for 15 min. The gel pieces were allowed to swell by incubating them with 20 mmol l^−1^ DTT in 0.1 mol l^−1^ NH_4_HCO_3_ for 45 min at 56 °C. After removing the DTT solution, the gel was incubated in 55 mmol l^−1^ iodoacetamide in 0.1 mol l^−1^ NH_4_HCO_3_ for 30 min in the dark. The gel was rinsed with 50 mmol l^−1^ NH_4_HCO_3_ and incubated in 50 mmol l^−1^ NH_4_HCO_3_/50% acetonitrile. After drying in a speedvac, an aliquot of 25 μg ml^−1^ sequencing-grade trypsin in 50 mmol l^−1^ NH_4_HCO_3_ was added. After a 45-min incubation on ice, the supernatant was discarded and replaced with 20 μl of 50 mmol l^−1^ NH_4_HCO_3_. Digestion was performed at 37 °C overnight, and fragmented peptides were extracted from the gel with 5% formic acid/50% acetonitrile. To improve the ionization efficiency of matrix-assisted laser desorption/ionization time-of-flight mass spectrometry (MALDI-TOF-MS), a ZipTipC18 column (Millipore) was used to purify peptides before MS analysis, according to the manufacturer's manual. The peptides were eluted with 2 μl of 5 mg ml^−1^ α-cyano-4-hydroxycinnamic acid in 50% acetonitrile/0.1% trifluoroacetic acid and applied directly onto the plate and allowed to air dry. Peptide mass fingerprints were obtained using a TOF-Spec 2E MALDI-TOF mass spectrophotometer (Waters). The Mascot program (Matrix Science) was used to interpret MS spectra of protein digests.

### Western blotting

Cells were treated as indicated in individual figure legends, and whole cell extracts were prepared in modified radioimmunoprecipitation assay (RIPA) buffer (Sigma) with the addition of protease and phosphatase inhibitors (Roche). Western blot analysis was performed and quantitated as described^52^. Proteins were separated by 10% SDS-PAGE and transferred to PVDF membranes (Bio-Rad Laboratories, Inc., Hercules, CA, USA). Dual colour precision protein MW markers (BioRad) were separated in parallel. Antibodies were purchased as follows: MVP (Cat#: 16478-1-AP) from ProteinTech, CD63 (Cat#: 143902) from Biolegend, Calnexin (Cat#: C45520) from Transduct. Lab, Caprin1 (Cat#: sc-83115), c-myc (Cat#: sc-41), cyclinD2 (Cat#: sc-181) and α-tubulin (Cat#: sc-8035) from Santa Cruz Biotechnology (Santa Cruz, CA, USA). The secondary antibodies conjugated to Fluors Alex-647 were purchased from Invitrogen (Eugene, OR, USA). The membranes were incubated with primary antibodies at dilution of 1:1,000 with PBST (PBS, 0.1% Tween 20) for 1 h at room temperature. After the secondary antibody incubation at dilution of 1:10,000 with PBST (PBS, 0.1% Tween 20) for 1 h at room temperature, the bands were visualized and analysed on an Odyssey Imager (LiCor Inc, Lincoln, NE, USA).

### Interaction of MVP and miR-193a assay

To further confirm the interaction of MVP and miR-193a, MVP was pulled down from CT26 cell lysates using anti-MVP antibody (Protein Tech) and protein G beads (ThermoFisher Scientific). Different concentrations of MVP were washed with buffer (PBS, 50 mM Tris-HCl, pH 7.5, 0.5 M NaCl, 0.1 mM EDTA, 1% Nonidet P-40, 0.5% sodium deoxycholate, 0.1% SDS, 1 mM sodium fluoride, 10 mg ml^−1^ phenylmethylsulfonyl fluoride, 2 μM aprotinin, 100 mM sodium orthovanadate) and were incubated with 2 μg of mature miR-193a in immunoprecipitation buffer (150 mM NaCl, 10 mM Tris-HCl (pH 7.4), 1 mM EDTA, 1 mM EGTA (pH 8.0), 0.2 mM sodium ortho-vanadate, 0.2 mM PMSF, 1% Triton X-100, 0.5% NP-40) for 2 h at 4 °C. The miR-193a/MVP complex was pulled-down, and the miR-193a remaining in the supernatant was collected for qPCR analysis.

### Histological analysis

Tissues were fixed with buffered 10% formalin solution (SF93-20; Fisher Scientific, Fair Lawn, NJ, USA) overnight at 4 °C. Dehydration was achieved by immersion in a graded ethanol series, that is, 70, 80, 95, 100% ethanol for 40 min each. Tissues were embedded in paraffin and subsequently cut into ultra-thin sections (5 μm) using a microtome. Deparaffinization was accomplished using xylene (Fisher) and rehydration using decreasing concentrations of ethanol and PBS. Tissue sections were stained with H&E and slides were scanned with an Aperio ScanScope. For tissue immunofluorescent staining, slides were washed three times (5 min each) with PBST. The tissue was permeabilized by incubating the slides in 1% Triton X-100 in PBS at 25 °C for 15 min and then washed three times in PBST. After blocking for 1 h at 25 °C in blocking buffer (PBS containing 10% BSA), slides were incubated overnight in a humid chamber with anti-MVP polyclonal antibody (ProteinTech). Antibodies were diluted 1:50 in blocking buffer. Following another three PBST washes, the slides were incubated with Alexa 647-conjugated secondary antibody at a 1:500 dilution (Invitrogen). The slides were then washed, and nuclei were counterstained with 4′,6-diamidino-2-phenylindole dihydrochloride.

For frozen sections, tissues were fixed with periodate-lysine-paraformaldehyde and dehydrated with 30% sucrose in PBS at 4 °C overnight. Tissue sections were stained with primary Ab in PBS/5% BSA (1:200) for 2 h and with secondary Ab in PBS/5% BSA (1:800) for 30 min. 4′,6-diamidino-2-phenylindole dihydrochloride was used as a nuclear stain.

### Gaussia luciferase (Gluc) activity assays

To test the Gluc activity of CT26 stably expressing EV-GlucB, 1 × 10^6^ CT26 cells were grown in exosome-free culture medium for 72 h. For the Gluc activity assay, 1 μg of exosomes from medium following treatment with RIPA lysis buffer (Sigma), 1 μg of whole cell lysates or 100 μl of medium was used. The activities of luciferase were determined after incubation with 50 μl of coelenterazine (CTZ) (10 ng ml^−1^, Nanolight) using the BioTek Synergy Microplate Reader. Values were averaged as indicated in the figure legends.

### Imaging of EV-GlucB distribution *in vivo*

To evaluate EV-GlucB bioluminescence activity in tumour-bearing mice, five BALB/c mice received an intrasplenic injection of CT26 cells stably expressing EV-GlucB. On day 14 after tumour cell inoculation, the mice were injected with 50 μl of CTZ (50 ng ml^−1^, Nanolight) or PBS as control, and bioluminescence activity was imaged 5 min after the injection using an Advanced Molecular Imager AMI (Spectral Instruments Imaging, AZ, USA) connected to an anaesthesia system (Summit, UT, USA).

### Electron microscopy of isolated exosomes

Isolated exosomes in PBS were fixed in 2% paraformaldehyde (Electron Microscopy Science, PA, USA) in PBS for 2 h at 22 °C followed by 1% glutaraldehyde (Electron Microscopy Science, PA, USA) for 30 min at 22 °C. Fixed samples (15 μl) were put on 2% agarose with formvar/carbon-coated nickel grids on top and allowed to absorb for 5–10 min. The grids with adherent exosomes were fixed in 2% paraformaldehyde in PBS for 10 min followed by extensive washing in PBS. Negative contrast staining was performed with 1.9% methyl cellulose and 0.3% uranyl acetate for 10 min. The grids with negatively stained exosomes were dried before observation under a Zeiss EM 900 electron microscope.

### Dot blot analysis of biotinylated exosomes

ExosomeGlucB stable expressive CT26 liver metastasis exosomes purified with streptavidin beads and exosomes from naïve liver were lysed with RIPA buffer. Lysate (1 μg) was spotted onto nitrocellulose membranes and blocked with 5% BSA in PBS at 4 °C overnight. CD63 and biotin were incubated with Alexa Fluor fluorescent conjugated anti-CD63 antibody (1:1,000) and streptavidin (1:1,000), respectively, at 4 °C overnight and then visualized.

### RNA interference

CT26 cells were grown to 70% confluency in six-well plates in antibiotic-free DMEM supplemented with 5% FBS. Cells were transfected with 90 pmoles miRNA or siRNA/well using 7 μl of RNAiMAX (Invitrogen) in antibiotic-free medium and incubated for 48–72 h. As a control, cells were transfected with scramble control miRNA or siRNA (Ambion). RNA and protein lysates were prepared for qPCR and western blot analysis.

### Knock-out (KO) and overexpression of MVP

Transduction of MVP sgRNA CRISPR lentiviral particles (>1 × 10^7^ IU ml^−1^) (GeneCopoeia, MD, USA) and MVP lentiviral activation particles (Santa Cruz, CA, USA) was utilized to KO and overexpress MVP in CT26 cells, respectively, according to the manufacturer's instructions. Briefly, 3 × 10^5^ cells in 1 ml of DMEM medium were seeded into the wells of a six-well culture plate. After 24 h, cells were infected by adding the lentiviral particles to the culture and incubated for 24 h. The medium was replaced with standard medium and incubated for two more days before analysis for mRNA and miRNA expression.

### Flow cytometric cell cycle analysis

Analysis of cell cycle distribution was carried out by flow cytometric analysis of propidium iodide (PI)-stained cells. The cells (1 × 10^6^) were fixed with 70% ethanol for 1 h at 20 °C. The samples were then centrifuged at 1,000*g* for 5 min. The 70% ethanol was removed, and the cells were then treated with 100 μl of RNase A (0.5 mg ml^−1^) for 30 min at 37 °C. Cell samples were then stained with 20 μg ml^−1^ of PI and analysed with a BD FACSCalibur flow cytometer to obtain DNA content profiles. FlowJo was used for analysis of the cell cycle.

### BrdU incorporation assay

BrdU was added to the culture medium for a final concentration of 3 μg ml^−1^ and incubated at 37 °C for 4 h. After the cells were fixed with 4% paraformaldehyde for 30 min, they were washed with PBS and treated with 2 N HCl to separate DNA into single strands. Cellular immunoreactivity for BrdU was visualized by FITC-conjugated anti-BrdU antibody (eBioscience, CA, USA) using a flow cytometer.

### Cell proliferation assay

Cell proliferation assays were performed using the Cell Titer96 AQueous One Solution Cell Proliferation Assay from Promega. Briefly, 1 × 10^3^ CT26 cells were plated per well in 96-well plates. The cells were treated with miRNA or siRNA indicated in the figure legends for 48 h to 5 days, depending on the experiment. Absorbance was measured at 490 nm using a SpectraMax M2 96-well plate reader (Molecular Devices, Sunnyvale, CA, USA). Each treatment was performed in quadruplicate within each experiment.

### Statistical analysis

All statistical analyses in this study were performed with SPSS 16.0 software. Data are presented as the mean±s.e. of the mean (s.e.m.). One-way analysis of variance followed by a Tukey *post-hoc* test was used to determine the differences occurring between more than two groups, and a *t*-test was used to determine the difference between two groups (*P<0.05, **P<0.01).

### Data availability

The data that support the findings of this study are available within the article and Supplementary Files, or available from the authors on reasonable request. The microarray data have been deposited in the Gene Expression Omnibus (GEO) under accession code GSE93092.

## Additional information

**How to cite this article:** Teng, Y. *et al*. MVP-mediated exosomal sorting of miR-193a promotes colon cancer progression. *Nat. Commun.* **8**, 14448 doi: 10.1038/ncomms14448 (2017).

**Publisher's note:** Springer Nature remains neutral with regard to jurisdictional claims in published maps and institutional affiliations.

## Supplementary Material

Supplementary Information Supplementary Figures and Supplementary Tables

## Figures and Tables

**Table 1:** List of significant higher level or lower level miRNAs presented in the tumour exosomes (fold change).

Higher in tumour exo	Primary cancer/Naïve (log2)	Metast/Primary cancer (log2)	Lower in tumour exo	Primary cancer/Naïve (log2)	Metast/Primary cancer (log2)
mmu-miR-10a-5p	2.17	5.22	mmu-miR-423-5p	−2.44	−10.21
mmu-miR-126a-3p	6.21	4.76	mmu-miR-301a-3p	−3.11	−10.04
mmu-miR-22-3p	8.01	4.19	mmu-miR-33-5p	−5.73	−7.22
mmu-miR-192-5p	4.11	4.11	mmu-miR-9-5p	−1.93	−5.92
mmu-miR-339-5p	4.51	4.01	mmu-miR-151-5p	−3.75	−5.20
mmu-miR-148a-3p	8.59	3.94	mmu-miR-196b-5p	−3.51	−5.05
mmu-miR-193a-3p	5.24	3.75	mmu-miR-147-3p	−3.83	−5.03
mmu-miR-30b-5p	2.73	3.74	mmu-let-7i-5p	−2.02	−4.99
mmu-miR-200b-5p	1.94	3.34	mmu-miR-1195	−2.30	−4.97
mmu-miR-677-5p	2.31	3.17	mmu-miR-96-5p	−3.41	−4.62
mmu-miR-154-3p	1.72	2.96	mmu-miR-669b-5p	−2.15	−4.60
mmu-miR-678	3.02	2.79	mmu-miR-380-3p	−2.36	−4.17
mmu-miR-222-3p	2.59	2.57	mmu-miR-196a-5p	−5.16	−4.02
mmu-miR-203-3p	6.86	2.52	mmu-miR-375-3p	−2.74	−3.99
mmu-miR-331-3p	3.13	2.44	mmu-miR-181d-5p	−3.32	−3.90
mmu-miR-714	12.01	2.25	mmu-miR-672-5p	−2.58	−3.74
mmu-miR-883b-3p	1.84	1.94	mmu-miR-654-3p	−2.43	−3.74
			mmu-miR-1199-5p	−1.74	−3.49
			mmu-miR-683	−1.80	−3.43
			mmu-miR-882	−1.94	−3.35
			mmu-miR-875-3p	−1.89	−3.29
			mmu-miR-711	−1.87	−3.20
			mmu-miR-182-5p	−2.45	−3.15
			mmu-miR-466f-5p	−2.23	−3.13
			mmu-miR-764-3p	−3.50	−2.85
			mmu-miR-504-5p	−2.16	−2.62
			mmu-miR-17-5p	−4.07	−2.61
			mmu-miR-1892	−1.38	−2.57
			mmu-miR-205-5p	−4.53	−2.51
			mmu-miR-146b-5p	−1.79	−2.46
			mmu-miR-301b-3p	−2.90	−2.16
			mmu-miR-15b-5p	−4.14	−1.92
			mmu-miR-450b-3p	−3.68	−1.74

**Table 2:** Differential distribution of miRNAs in exosomes and exosomes donor cells (fold change).

**Higher in tumour exo**	**Exosomes/tissue (log2)**			**Biological effect**	**Lower in tumour exo**	**Exosomes/tissue (log2)**			**Biological effect**
	**Naïve**	**Primary cancer**	**Metastasis**			**Naïve**	**Primary cancer**	**Metastasis**	
mmu-miR-10a-5p	−4.54	−3.22	−2.87	TS/Onco	mmu-miR-423-5p	−0.07	−0.42	−7.49	Onco
mmu-miR-126a-3p	1.88	−0.41	−2.35	TS/Once	mmu-miR-301a-3p	4.41	3.25	2.97	Onco
mmu-miR-22-3p	−12.27	−8.25	−0.05	TS	mmu-miR-33-5p	1.87	1.31	1.24	Onco/TS
mmu-miR-192-5p	−6.07	−4.07	−2.74	TS	mmu-miR-9-5p	1.10	−1.15	−3.78	TS
mmu-miR-339-5p	−7.11	−3.19	0.42	TS	mmu-miR-151-5p	1.02	4.98	7.91	Onco
mmu-miR-148a-3p	−13.07	−3.34	−0.70	TS	mmu-miR-196b-5p	8.99	−6.74	−10.65	Onco
mmu-miR-193a-3p	−7.01	2.58	7.77	TS	mmu-miR-147-3p	−0.48	−1.12	−1.97	TS
mmu-miR-30b-5p	−6.27	−2.21	−1.56	TS	mmu-let-7i-5p	0.25	3.36	5.10	TS
mmu-miR-200b-5p	−9.94	−5.57	−4.53	TS	mmu-miR-1195	−0.30	0.15	0.46	—
mmu-miR-677-5p	0.49	−1.05	7.76	—	mmu-miR-96-5p	1.43	−1.10	−0.90	Onco
mmu-miR-154-3p	−3.93	2.01	5.43	TS	mmu-miR-669b-5p	0.31	−2.57	−5.07	—
mmu-miR-678	−5.82	−1.09	−1.76	—	mmu-miR-380-3p	−0.07	−1.88	−3.46	Onco
mmu-miR-222-3p	−2.42	3.64	8.62	TS/Onco	mmu-miR-196a-5p	7.97	5.36	3.78	Onco
mmu-miR-203-3p	0.03	2.24	−1.65	TS	mmu-miR-375-3p	17.09	11.17	9.70	Onco
mmu-miR-331-3p	−5.17	−2.87	−1.78	TS	mmu-miR-181d-5p	0.45	−3.05	−4.81	Onco
mmu-miR-714	4.71	5.38	9.67	—	mmu-miR-672-5p	7.38	5.47	5.65	—
mmu-miR-883b-3p	0.95	−4.49	−7.50	—	mmu-miR-654-3p	5.88	−2.97	−5.51	—
					mmu-miR-1199-5p	2.32	−0.44	−1.69	—
					mmu-miR-683	0.32	−1.69	−3.88	—
					mmu-miR-882	−0.61	−2.23	−1.87	—
					mmu-miR-875-3p	2.42	1.14	0.35	—
					mmu-miR-711	1.21	−4.46	−6.21	Onco
					mmu-miR-182-5p	0.13	−1.07	−1.81	Onco
					mmu-miR-466f-5p	−0.49	1.12	−2.74	—
					mmu-miR-764-3p	2.50	−1.08	−3.64	—
					mmu-miR-504-5p	6.17	−1.32	1.77	TS
					mmu-miR-17-5p	0.17	−3.71	−7.81	Onco
					mmu-miR-1892	1.39	−4.07	−8.27	—
					mmu-miR-205-5p	3.82	−2.84	−1.04	Onco/TS
					mmu-miR-146b-5p	0.01	−1.12	−2.30	Onco
					mmu-miR-301b-3p	0.99	−2.87	−3.56	Onco
					mmu-miR-15b-5p	0.64	−2.30	−3.02	Onco
					mmu-miR-450b-3p	−4.20	−3.21	−3.50	—

Onco, oncogenic miRNAs; TS, tumour suppressive miRNAs; —, unknown.
