# Supplementary material for: MVP-mediated exosomal sorting of miR-193a promotes colon cancer progression
Source: Nat Commun. 2017 Feb 17;8:14448. doi: 10.1038/ncomms14448 (PMC5321731; doi:10.1038/ncomms14448)
Supplement: Supplementary Information — Supplementary Figures and Supplementary Tables [file ncomms14448-s1.pdf]

Supplementary Figures

Supplementary Figure 1

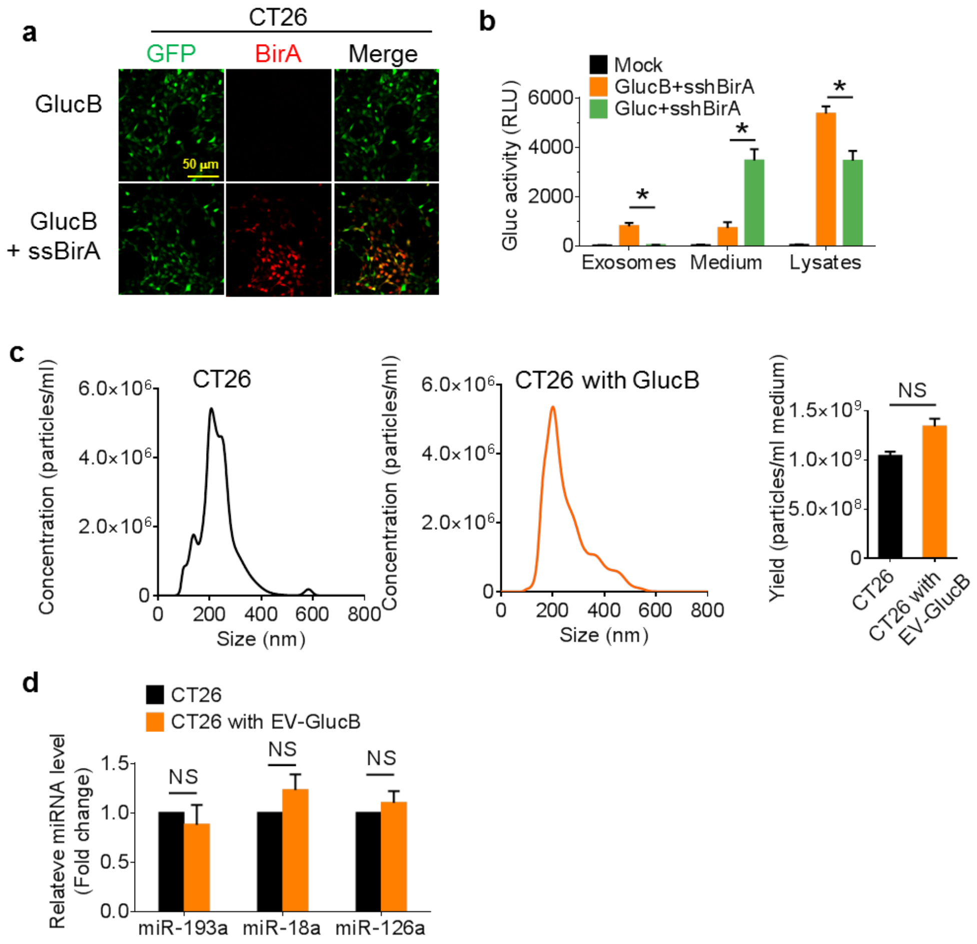

Supplementary Figure 1 Characterization of stable expression of GlucB and sshBirA in the CT26 cell line (**a**) Live cell imaging of stable CT26 cells expressing green fluorescent protein (GFP) conjugated membrane-bound *Gaussia* luciferase (GlucB) with or without biotin ligase (BirA). Scale bars, 50 µm. (**b**) Activity of reporter using Gluc luciferase assay with Gluc substrate coelentrazine (CTZ) in the exosomes from the stable CT26 cell line, medium and cell lysates. RLU; relative light units. (**c**) Size distribution (left, middle) and yield (right) of exosomes from culture supernatant of CT26 with or without GlucB. (**d**) Analysis of selected miRNAs level in exosomes from culture supernatant of CT26 with or without GlucB using qPCR. Data are representative of three independent experiments. *P < 0.05 (two-tailed t-test; error bars, S.E.M.); NS represents non-significance.

Supplementary Figure 2

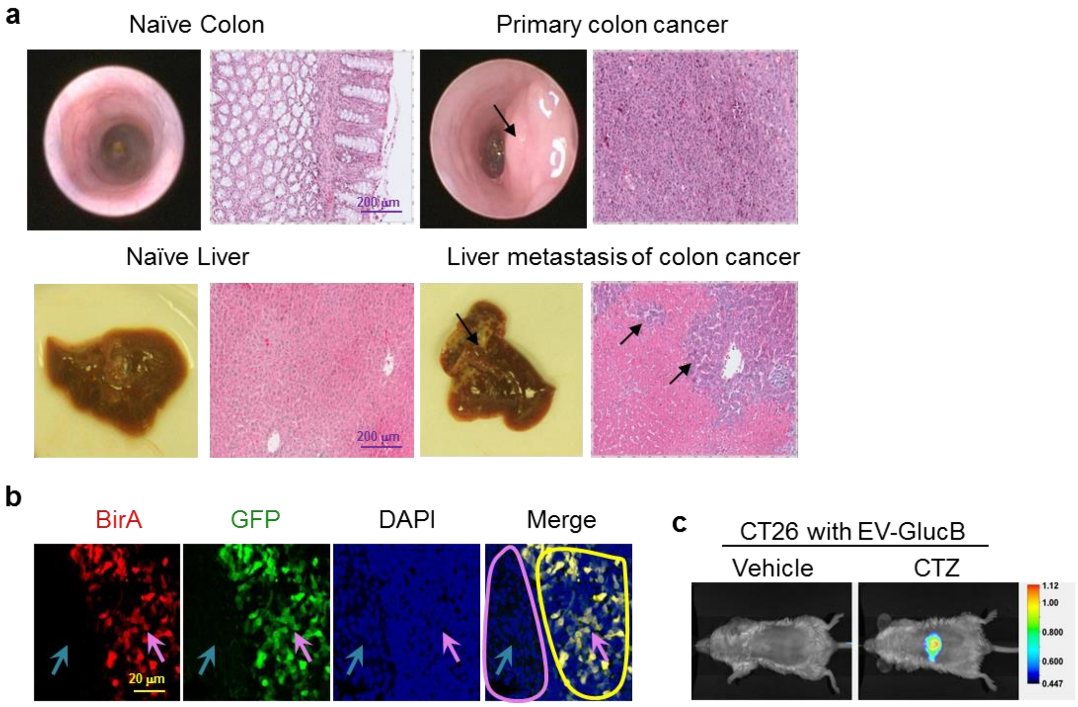

Supplementary Figure 2 An in vivo expression of GlucB and sshBirA in the stable CT26 cells (**a**) Representative endoscopic view of mouse colon and hematoxylin and eosin (H&E)-stained sections of colon tissue (up panel). 1x10^6 stable EV-GlucB expressing CT26 cells (up panel, third and fourth columns) administered to BALB/c mice (n=5 each group) via colon submucosal (up right) or intra-splenic injection (bottom panel, third and fourth column). Representative colon (up panel), liver (bottom panel), and representative H&E-stained sections of formalin-fixed, paraffin-embedded colon and liver (400x magnification, Scale bars, 200 μm) from naive BALB/c and tumor bearing mice at 14 d after administration of CT26 cells are shown. (**b**) Visualization by confocal microscopy in sectioned liver of CT26 cells stably expressing GlucB-GFP and BirA . Arrows in pink indicate tumor metastasis in liver; Arrows in blue indicated adjacent normal liver tissue; Scale bars, 20 μm. Metastatic liver of CT26 was collected from BALB/c mice 2 weeks after intra-splenic injection of 1x10^6 CT26 cells. (**c**) *In vivo* imaging of EV-GlucB expression in CT26 cells. BALB/c mice (n=5) were administered vehicle (control) or CTZ by intravenous injection 2 weeks after intra-splenic injection of 1x10^6 stable CT26 cells.

Supplementary Figure 3

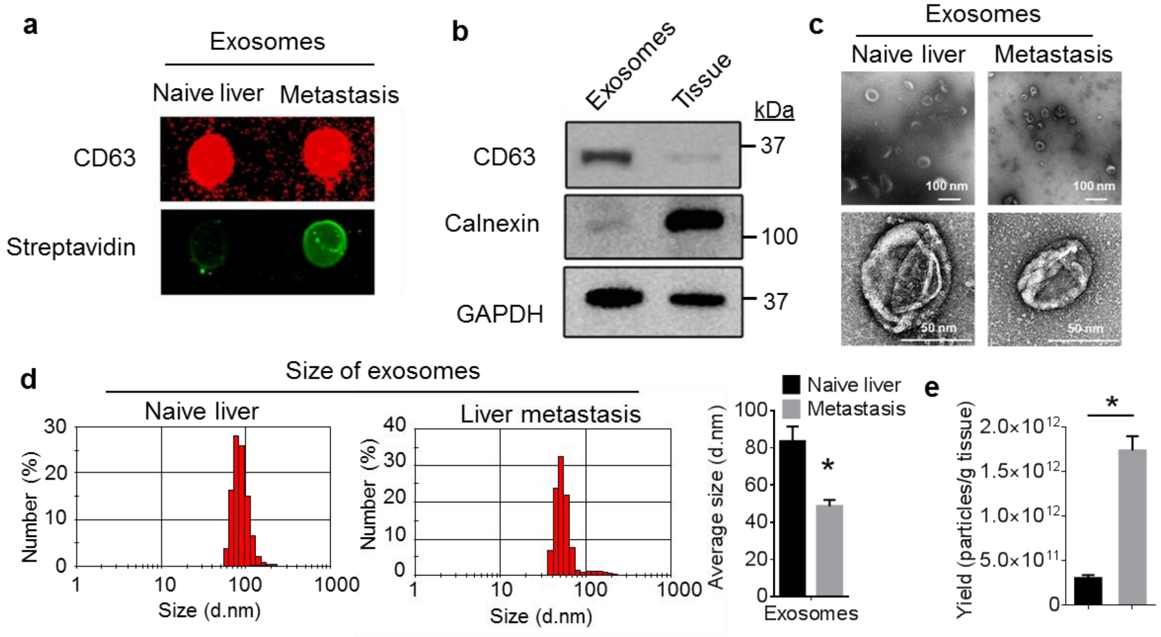

Supplementary Figure 3 Purification and characterization of exosomes from liver metastasis of colon cancer using EV-GlucB reporter (**a**) Dot blot detection of EV-GlucB using exosomal marker CD63 antibody and streptavidin. Exosomes were isolated from naïve liver and liver metastatic CT26 cell line which was stably expressing GlucB and BirA. (**b**) Expression of CD63 and endoplasmic reticulum protein Calnexin in exosomes and tissue from liver metastasis of colon cancer assessed by western blot. (**c**) Representative electron microscopic image of exosomes from liver metastasis of colon cancer and naïve liver. Scale bars, 100 nm (top); Scale bars, 50 nm (bottom). (**d**) Size distribution of exosomes from naïve liver (left panel) or liver metastasis of colon cancer (middle panel) analyzed using a Zetasizer Nano ZS. Quantification of average size (right panel) and yield (**e**) of exosomes from naïve liver and liver metastasis of colon cancer. **P* < 0.05 (two-tailed t-test). Data are representative of three independent experiments (error bars, S.E.M.).

Supplementary Figure 4

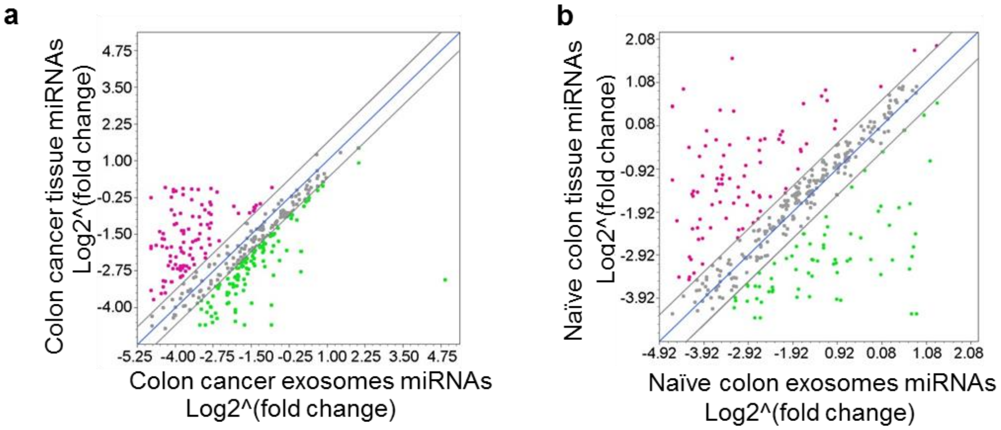

Supplementary Figure 4 Microarray data visualization by scatter plot comparing exosomes miRNAs with donor tissues miRNAs Microarray data visualization by scatter plot comparing exosome miRNAs (X-axis) with donor tissue miRNAs (Y-axis) in colon cancer (**a**) and naïve colon (**b**) (Differential expression of log2 value > 2). In the scatter plot each point represents the expression value of a gene, the green and red dots represent the genes highly expressed in exosomes and tissues, respectively, and the grey dots represent similar gene expression in exosomes and tissues.

Supplementary Figure 5

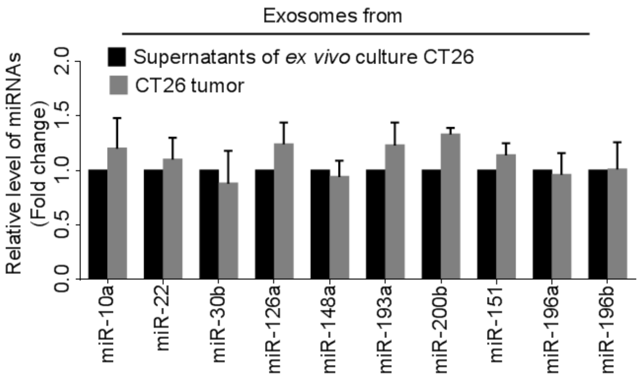

Supplementary Figure 5 qPCR analysis of selected miRNAs level in exosomes from the culture supernatant of 12 h cultured primary colon cancer cells and exosomes from primary colon cancer tissue.

Supplementary Figure 6

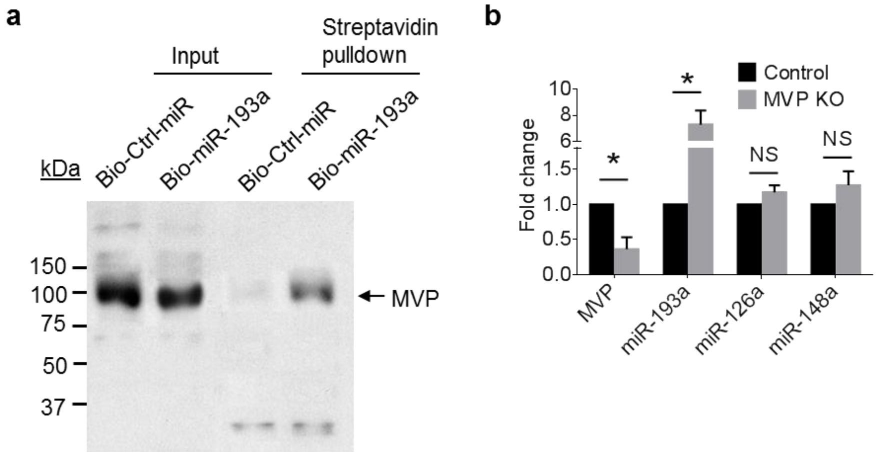

Supplementary Figure 6 Specific interaction of miR-193a and MVP (**a**) Western blot analysis expression of MVP before (Input) and after streptavidin pulldown of lysates of CT26 cells transfected with Bio-miR-193a or control miRNA (Bio-Ctrl-miR). (**b**) MVP KO cells were generated using the CRSPR/Cas9 system. Selected miRNAs, as well as MVP expression, in cell lysates were evaluated by qPCR. *P < 0.05 (two-tailed t-test); NS represents non-significance. Data are representative of three independent experiments (error bars, S.E.M.).

Supplementary Figure 7

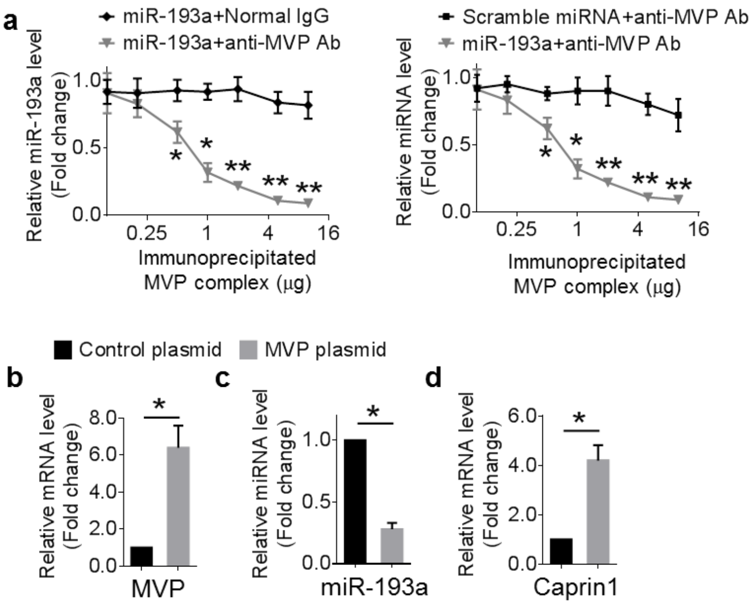

Supplementary Figure 7 MVP competes with miR-193a for binding and suppressing Caprin1 expression (**a**) 2 μg of miR-193a incubated with MVP and miR-193a/MVP complex has been pulled-down with anti-MVP antibody. qPCR analysis of miR-193a or RNA quantitation in the supernatant after depletion of miR-193a/MVP complex. (**b-d**) qPCR analysis of MVP (**b**), miR-193a (**c**), and Caprin1 (**d**) expression in CT26 cells transfected with MVP lentiviral activation particles. *P < 0.05 and **P < 0.01 (two-tailed t-test). Data are representative of three independent experiments (error bars, S.E.M.).

Supplementary Table 1: Primer sequences used for quantitative Real-Time PCR (qPCR) of mRNA

Primers		Forward (5'-3')	Reverse (5'-3')
qPCR	MVP	AGACGAGTGGCTGTTTGAG	CAGAGCTTGGTTCTGTTTGATG
	CAPRIN1	CTTATGGCACAAATGCAAGGG	CATGTTCTGGGTAGGGTTCATAG
	CCND2	GAAGGACATCCAACCGTACAT	TTCATGGCCAGAGGAAAGAC
	C-MYC	GCGATCAGCTCTCCTGAAA	GCAGAAAGAACACAGGGAAAG
	GAPDH	GGTCGGTGTGAACGGATTTG	GGAGTCATACTGGAACATGTAG
Mutant	Mutantgenesis	GTGTTTTTGGCGATTAAACATAATCC TG	ACTGACTCGTGTACCATAATATG TTACCAG
